# Distinct glutamatergic projections of the posteroventral medial amygdala play different roles in arousal and anxiety

**DOI:** 10.1172/jci.insight.176329

**Published:** 2024-06-06

**Authors:** Ying Li, Yuchen Deng, Yifei Zhang, Dan Xu, Xuefen Zhang, Yue Li, Yidan Li, Ming Chen, Yuxin Wang, Jiyan Zhang, Like Wang, Yufeng Cang, Peng Cao, Linlin Bi, Haibo Xu

**Affiliations:** 1 Department of Radiology, Zhongnan Hospital of Wuhan University, Wuhan University, Wuhan, China.; 2 Department of Pathology, Taikang Medical School (School of Basic Medical Sciences), Wuhan University, Wuhan, China.; 3 Center for Pathology and Molecular Diagnostics,; 4 Department of Nuclear Medicine, and; 5 Department of Cardiology, Zhongnan Hospital of Wuhan University, Wuhan University, Wuhan, China.; 6 National Institute of Biological Sciences, Beijing, China.; 7 Tsinghua Institute of Multidisciplinary Biomedical Research, Tsinghua University, Beijing, China.; 8 Guangdong Province Key Laboratory of Psychiatric Disorders, Southern Medical University, Guangzhou, China.

**Keywords:** Neuroscience, Neurological disorders

## Abstract

Sleep disturbance usually accompanies anxiety disorders and exacerbates their incidence rates. The precise circuit mechanisms remain poorly understood. Here, we found that glutamatergic neurons in the posteroventral medial amygdala (MePV^Glu^ neurons) are involved in arousal and anxiety-like behaviors. Excitation of MePV^Glu^ neurons not only promoted wakefulness but also increased anxiety-like behaviors. Different projections of MePV^Glu^ neurons played various roles in regulating anxiety-like behaviors and sleep-wakefulness. MePV^Glu^ neurons promoted wakefulness through the MePV^Glu^/posteromedial cortical amygdaloid area (PMCo) pathway and the MePV^Glu^/bed nucleus of the stria terminals (BNST) pathway. In contrast, MePV^Glu^ neurons increased anxiety-like behaviors through the MePV^Glu^/ventromedial hypothalamus (VMH) pathway. Chronic sleep disturbance increased anxiety levels and reduced reparative sleep, accompanied by the enhanced excitability of MePV^Glu^/PMCo and MePV^Glu^/VMH circuits but suppressed responses of glutamatergic neurons in the BNST. Inhibition of the MePV^Glu^ neurons could rescue chronic sleep deprivation–induced phenotypes. Our findings provide important circuit mechanisms for chronic sleep disturbance–induced hyperarousal response and obsessive anxiety-like behavior and are expected to provide a promising strategy for treating sleep-related psychiatric disorders and insomnia.

## Abstract 2

Amygdala-related circuit mechanisms are linked to abnormal sleep architecture and anxiety disorders.

## Graphical abstract

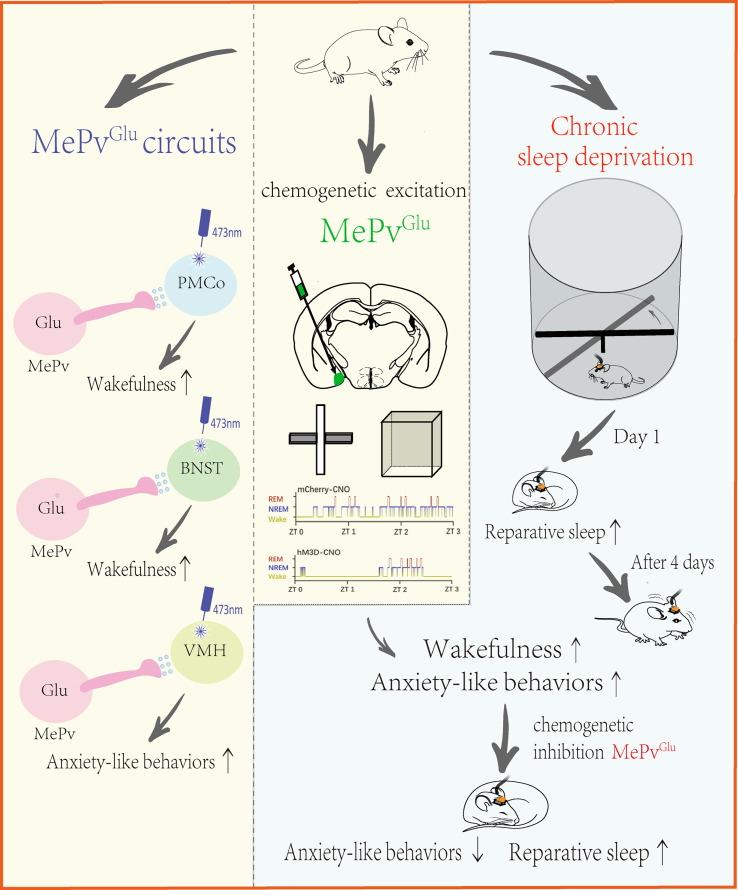

## Introduction

Moderate vigilance and fear in the face of threats can help animals quickly detect danger and wake up from sleep. However, hyperarousal response and obsessive anxiety-like behavior are involved in several adverse sleep architectural changes (1, 2). Stress, such as a physical or emotional stimulus, can cause disrupted or shortened sleep (3, 4). Some patients with anxiety disorders often exhibit hyperarousal responses to sensory stimuli (5). Furthermore, the presence of sleep disturbances has been found to exacerbate the risk of developing anxiety disorders (1, 6). Passive sleep deprivation could worsen anxiety disorders and cause changes in the structure of sleep, but little research has been done on the mechanisms by which sleep deprivation impairs the emotion and sleep systems. Elucidating the neural circuits that control physiological arousal to detect threats is imperative to understanding maladaptive emotional states and abnormal sleep changes.

The medial amygdala (Me) is a critical brain region that encodes fear-related behavior (7, 8). The Me is composed of several subnuclei with differing structures and functions, such as the posterodorsal (MePD), posteroventral (MePV), and anterior (MeA) (9–11). Most MePD neurons are GABAergic, whereas many MeA/PV ones are glutamatergic (9–11). MePV cells that project to the ventromedial hypothalamus (VMH) are glutamatergic neurons and increase their activity in response to cat odor in mice (12). Previous studies have shown that MePV and paraventricular hypothalamic nucleus (PVN) upregulated c-fos expression when rats were exposed to cat odor (13). In the present study, we focused on the function of the MePV.

The MePV was implicated in coping with inherent danger cues by activity mapping of the immediate-early gene c-fos (12, 14). In mice, the MePV is triggered when a natural danger stimulus approaches (15). Distinct subpopulations of MePV neurons project to the bed nucleus of the stria terminals (BNST) and the VMH in different ways. These projections have opposing consequences on investigating or avoiding potentially dangerous stimuli (15). Previous research demonstrated that VMH outputs play a role in modulating anxiety-like behavior and fear (16). Results from an open field experiment (8) revealed that VMH neurons expressing the nuclear receptor protein NR5A1 (also known as SF1) in mice displayed sustained activity encountering stimuli necessary for persistent protective behavior, indicating that MePV-VMH has a putative function in controlling anxiety-like behaviors. Although BNST is involved in the modulation of anxiety-like behaviors and fast changes in arousal (17), the effect of projecting MePV neurons to the BNST on the regulation of arousal or anxiety remains unclear.

A previous study suggested that the posteromedial cortical amygdaloid area (PMCo) might regulate social odor and primary olfactory processing (18). Although the authors revealed the connectivity of the PMCo feedback circuit innervating the mouse accessory olfactory bulb (18–20), the functions of PMCo on wakefulness-sleep or anxiety were barely reported. Although our preliminary data revealed an increase in PMCo activity by the chemogenetic excitation of MePV neurons, the role of MePV/PMCo in wake-sleep or anxiety was unknown. Therefore, we hypothesized that the MePV/PMCo, MePV/VMH, and MePV/BNST pathway might be important gates for controlling responses to threatening stimuli or physiological arousal. Further research exploration is needed to elucidate cell type– and circuit-specific features in the MePV that control the integration of stress and sleep-wake.

Previous research has shown that glutamatergic neurons in other brain areas, such as the PVN, can control anxiety and arousal (21). Given the MePV’s role in regulating innate defensive responses, coupled with the facts that MePV neurons are mainly glutamatergic (9, 11, 17) and that downstream brain region BNST is involved in arousal behaviors, we hypothesized that glutamatergic neurons in the MePV are potentially involved in regulating anxiety-like behaviors and arousal response to threatening stimuli. In the present study, we employed in vivo calcium imaging and cell type–specific manipulations to reveal that neural circuits involving MePV^Glu^ neurons not only regulate both anxiety-like behaviors and sleep-wakefulness but also control sleep deprivation–mediated obsessive anxiety-like behavior and sleep change. We also investigated the downstream neural networks and underlying processes for modulating responses to hazardous stimuli or physiological arousal.

## Results

### MePV^Glu^ neurons are selectively active during threat-evoked wake from non-rapid eye movement sleep.

**Figure 1:**
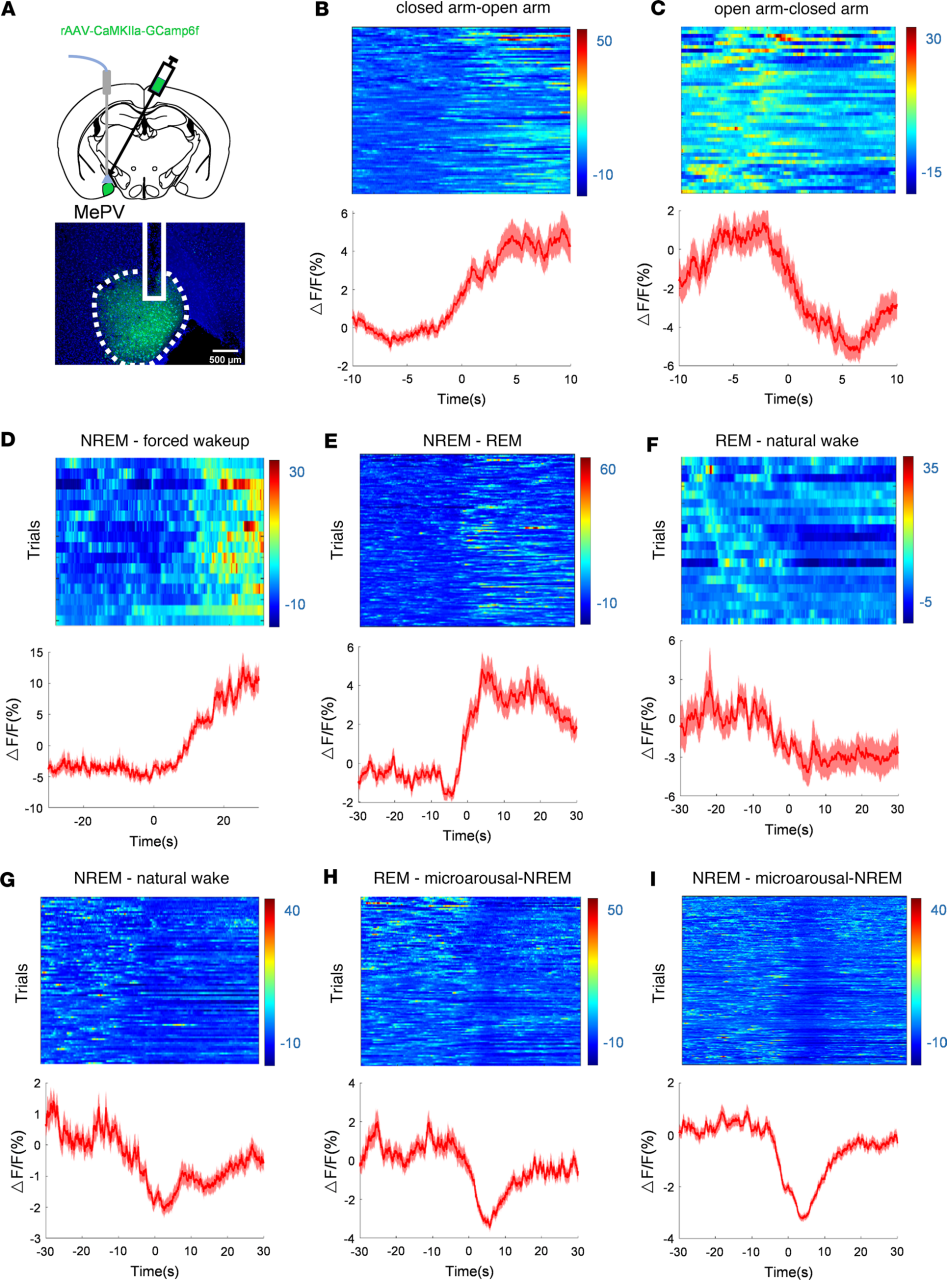
The effects of NREM-to-REM transitions, NREM-to-unusual wake transitions, and REM-to-wake transitions on the activity of MePV^Glu^ neurons. (**A**) Top: The in vivo recording arrangement; Bottom: Illustrations of the MePV from a mouse expressing the rAAV-CaMKIIa-GCaMP6f virus (*n* = 6 mice/group), displaying virus expression as well as the position of the fiber tip above the MePV. (**B**) The heatmap for the calcium signal of MePV^Glu^ neurons. The representative transitions (closed arm-open arm, 87 trials) of the changes in color-coded fluorescence intensity. (**C**) The color-coded fluorescence intensity changes of the representative shifts from the open arm to the closed arm (45 trials). (**D**) A shift in color-coded fluorescence intensity illustrating the representative transition from NREM to forced wakeup (16 trials). (**E**) Changes in color-coded fluorescence intensity indicating the representative transitions from NREM to REM (132 trials). (**F**) The representative shifts in fluorescence intensity during the transitions from REM to normal wakefulness (20 trials). (**G**) Representative shift in color-coded fluorescence intensity (83 trials) depicting the transition from NREM to natural wake. (**H**) Color changes representing shifts in fluorescence intensity from REM to microarousal (119 trials). (**I**) A shift in the intensity of the color-coded fluorescence indicating the representative transition from NREM to microarousal (257 trials). Mean (red trace) ± SEM (red shading) represents the average responses of all the transitions.

To check whether endogenous glutamatergic neurons in the MEPV (MePV^Glu^ neurons) are involved in the regulation of the natural sleep-wake cycle or anxiety-like behaviors, we injected AAV-CaMKIIa-GCaMP6f into the MePV of C57BL/6J mice (MePV^Glu^-GCaMP6f mice) and recorded changes in Ca^2+^ signal during sleep state transitions using the electroencephalogram and electromyogram (EEG-EMG) system or anxiety state transitions during behavioral tests (Figure 1A and Supplemental Figure 1; supplemental material available online with this article; https://doi.org/10.1172/jci.insight.176329DS1). On the elevated plus maze test, the level of Ca^2+^ signal increased when mice approached the open arms and dropped when mice returned to the closed arms from the open arms (Figure 1, B and C, and Supplemental Figure 1, G and H). Subsequently, the loud noise of opening the soundproof door of the recording chamber was used to simulate the intruder’s stimulus while capturing the Ca^2+^ signal (to make this experiment consistent every time, the noises of 25–30 dB measured by a sound decibel meter were selected). The Ca^2+^ signal increased dramatically from non-rapid eye movement (NREM) to threat-evoked waking and from NREM to rapid eye movement (REM) sleep (Figure 1, D and E, and Supplemental Figure 1, A and B). In addition to this, we explored the alterations of neuronal Ca^2+^ signals in mice that were forced to wake up under other stimulating conditions (Supplemental Figure 2, B and C). It was found that the ΔF/F ratio increased from NREM to threat-evoked wake and from NREM to REM (Figure 1, D and E) but dropped from REM to natural wake (Figure 1F). The ΔF/F ratio increased when mice were forced to wake up with alcohol spraying (Supplemental Figure 2B) or 90 db white noise stimulation (Supplemental Figure 2C). The results showed that the Ca^2+^ signal of MePV^Glu^ neurons changed significantly when they were forced to wake up. The change in ΔF/F ratio was minimal throughout the transition from NREM to natural wake (Figure 1G). During the microarousal stage, which occurred during sleep, the ΔF/F ratio decreased (Figure 1, H and I). Endogenous MePV^Glu^ neurons were found to respond to threatening situations, threat-evoked wakefulness, and microarousal but not in natural wakefulness.

### Chemogenetic excitation of MePV^Glu^ neurons promotes wakefulness and increases anxiety-like behaviors.

**Figure 2:**
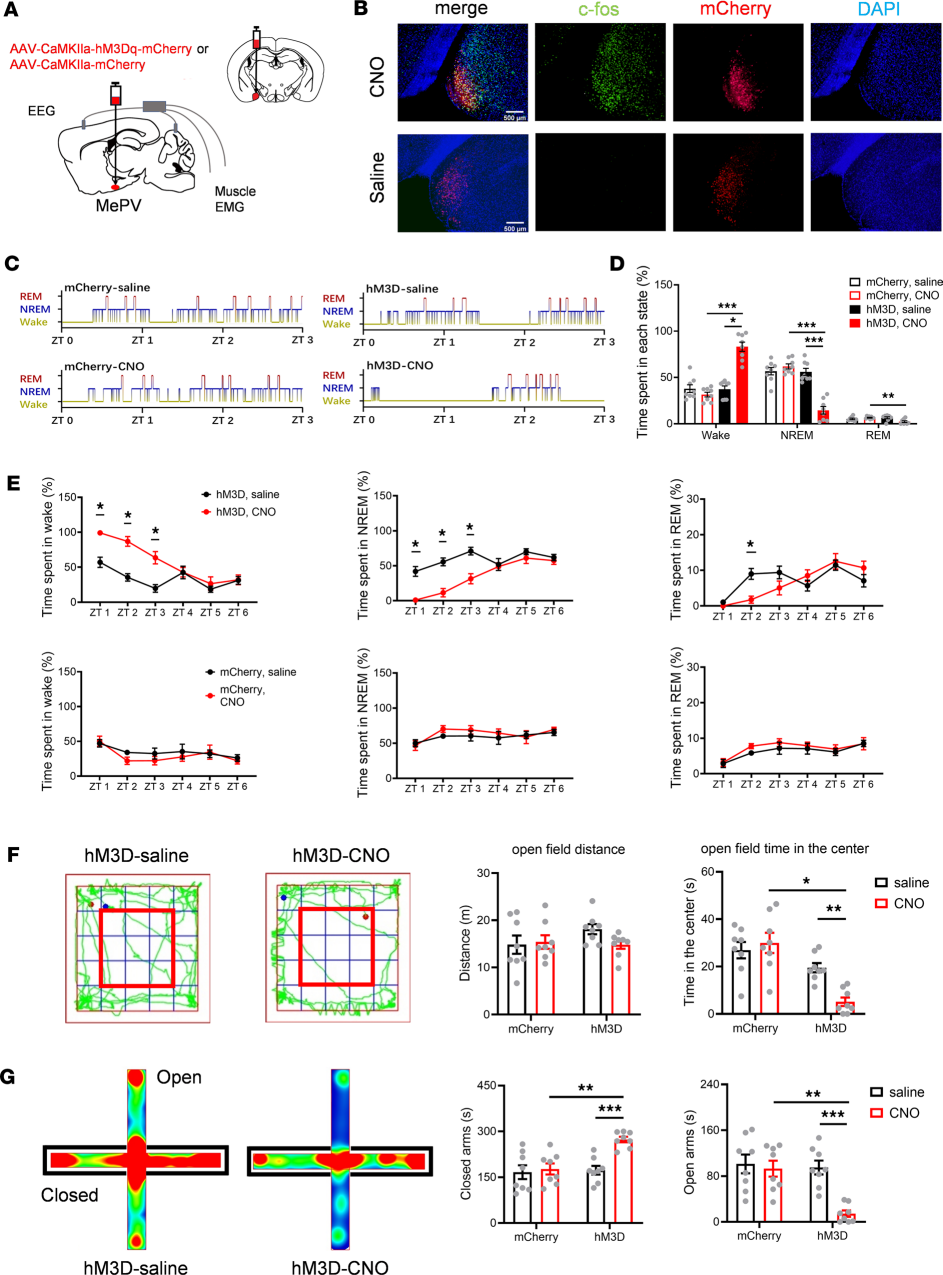
The effects of chemogenetic excitation of MePV^Glu^ neurons on wakefulness and anxiety-like behavior. (**A**) Schematic diagram showing AAV-CaMKIIa-hM3Dq-mCherry/AAV-CaMKIIa-mCherry virus injection and EEG-EMG recordings. (**B**) Representative images (original magnification, ×10) of c-fos (green), mCherry (red), and DAPI (blue) colocalization in MePV^Glu^ neurons of MePV^Glu^-hM3Dq-mCherry mice treated with CNO or saline. Scale bar = 500 μm. (**C**) Three-hour hypnograms following saline or CNO (1 mg/kg) injections into an MePV^Glu^-mCherry mouse (left) and an MePV^Glu^-hM3Dq-mCherry mouse (right). (**D**) Percentages of time spent in each state for MePV^Glu^-hM3Dq-mCherry mice and MePV^Glu^-mCherry mice 3 hours after CNO injection. (**E**) Time spent in each condition 6 hours after injection of saline or CNO (1 mg/kg) into the MePV^Glu^-hM3Dq-mCherry mice (top) and the MePV^Glu^-mCherry mice (bottom). (**F**) Left panel: Representative track plots of MePV^Glu^-hM3Dq-mCherry mice treated with saline and CNO in open field test (red frame represents the central zone). Right panel: Time spent in the center zone and distance traveled of MePV^Glu^-hM3Dq-mCherry mice and MePV^Glu^-mCherry mice. (**G**) Left panel: Representative heatmaps of MePV^Glu^-hM3Dq-mCherry mice treated with saline and CNO in elevated plus maze (closed arms on black frames). Right panel: Time spent in open/closed arms of MePV^Glu^-hM3Dq-mCherry mice and MePV^Glu^-mCherry mice. Wilcoxon signed rank test or 2-way repeated measures (RM) ANOVA test with Holm-Šídák post hoc comparison for **D** and **E**. Two-way RM ANOVA test with Holm-Šídák post hoc for **F** and **G**. *n* = 8 per group. *P < 0.05, **P < 0.01, ***P < 0.001.

Although endogenous MePV^Glu^ neurons may not be involved in calm sleep and wakefulness, overexcited MePV^Glu^ neurons can potentially be involved in stress-induced hyperarousal, as suggested by the above research. However, whether the increased Ca^2+^ signal of MePV^Glu^ neurons when mice entered the open arms triggered obsessive anxiety-like behavior or exploration-related behaviors was still uncertain. Using the bilateral expression of excitatory hM3Dq receptors that were specifically induced by clozapine-N-oxide (CNO), the behavioral effect of chemogenetic activation of MePV^Glu^ neurons was examined. Three weeks after the viral injection, a histological examination was conducted to confirm hM3Dq-mCherry expression, particularly in MePV^Glu^ neurons (Figure 2A).

We first demonstrated that MePV^Glu^ neurons in the MePV^Glu^-hM3Dq mice were excited by CNO. One hour after CNO or saline i.p. injection into MePV^Glu^-hM3Dq mice, activated neurons (c-fos–positive) were significantly increased in the CNO group compared with those in the control group (Figure 2B and Supplemental Figure 3). After the CNO treatment (1 mg/kg, i.p. injection), the wakefulness remained elevated for 3 hours. Both NREM sleep and REM sleep were decreased by the CNO treatment (Figure 2, C–E). As a control for the specificity of CNO’s actions, we injected AAV-CaMKIIa-mCherry into the MePV of C57BL/6J mice; CNO injection (i.p.) into these MePV^Glu^-mCherry mice did not affect sleep or wakefulness time (Figure 2, C–E). Typical examples of hypnogram, EMG track, EEG track, and EEG spectrogram for 3 hours from MePV^Glu^-hM3Dq mice respectively given saline or CNO (i.p., 1 mg/kg) are shown in Supplemental Figure 8, A and B.

Stimulation of MePV^Glu^ neurons did not result in hyperactivity. Moreover, the locomotion of CNO-injected mice was not significantly different from that of saline-injected mice in the open field test. Further, chemogenetic stimulation of MePV^Glu^ neurons reduced the time spent in the center in the open field test (Figure 2F). The CNO injection increased the time spent on the closed arms while decreasing the time spent on the open arms on the elevated plus maze (Figure 2G). Overall, these results suggest that chemogenetic stimulation of MePV^Glu^ neurons might increase wakefulness and anxiety-like behaviors but does not influence locomotion.

To test whether the MePV functions of female mice and male mice were different, we injected AAV-CaMKII-hM3Dq-mCherry virus and AAV-CaMKII-mCherry virus into MePV^Glu^ neurons of female mice and implanted EEG-EMG electrodes (Supplemental Figure 4A). After 3 weeks of recovery, same as males, NREM sleep of females was decreased by the CNO treatment (Supplemental Figure 4B). The CNO injection increased the time spent on the closed arms while decreasing the time spent on the open arms on the elevated plus maze (Supplemental Figure 4, C and E). Chemogenetic stimulation of MePV^Glu^ neurons slightly reduced the time spent in the center in the open field test (Supplemental Figure 4, D and F). Overall, these results suggested that MePV^Glu^ neurons played a similar role in regulating sleep and anxiety-like behaviors in both female and male mice.

### Optogenetic excitation of MePV^Glu^ neurons promoted wakefulness and increased anxiety-like behaviors.

To confirm that mice relied on MePV^Glu^ neurons to regulate anxiety-related behaviors or to govern arousal behaviors during NREM sleep and waking states, we next tested whether optogenetic activation of MePV^Glu^-Channelrhodopsin 2–expressing (MePV^Glu^-ChR2–expressing) neurons influenced wakefulness and anxiety-like behavior.

**Figure 3:**
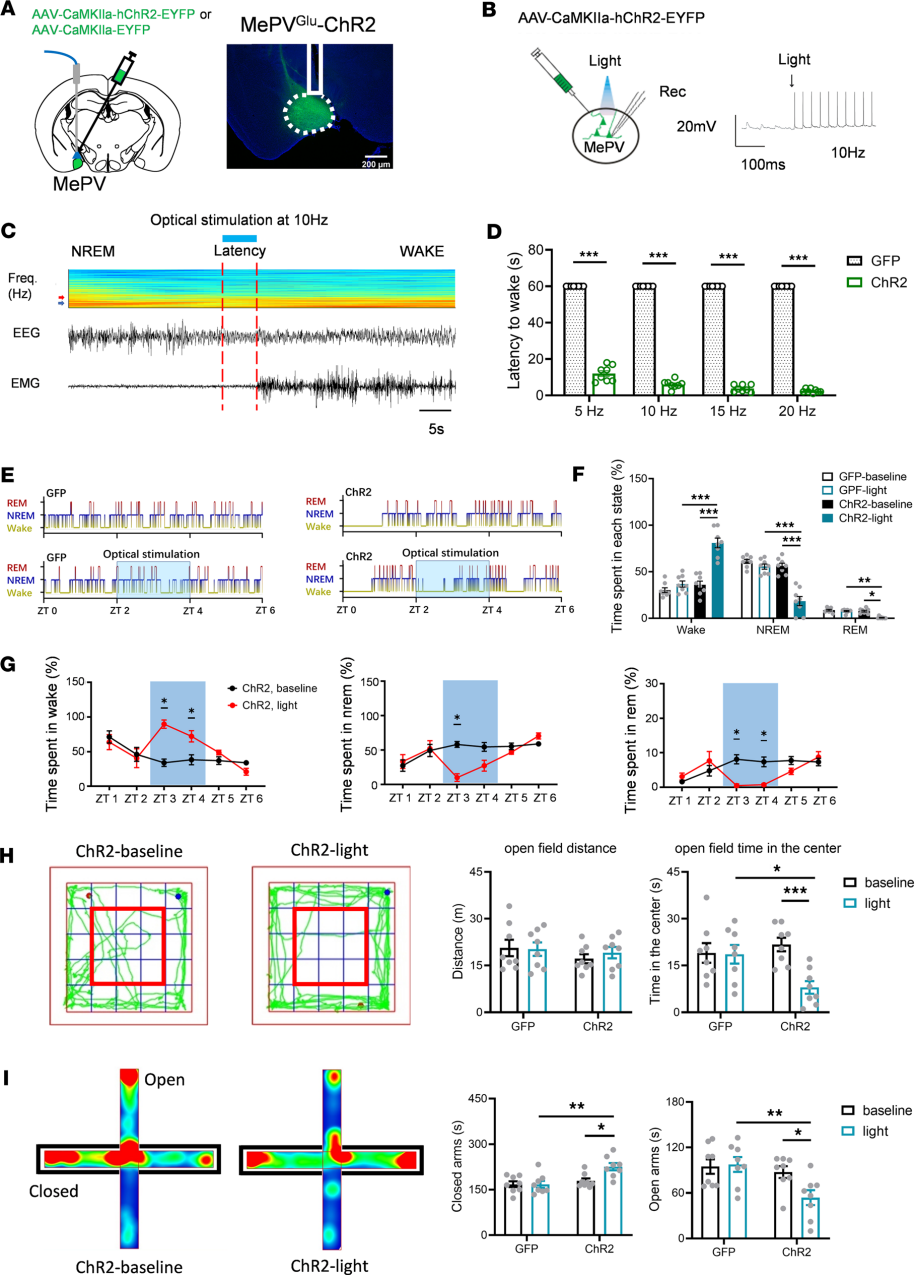
The effects of optogenetic stimulation of MePV^Glu^ neurons on wakefulness and anxiety-like behavior. (**A**) Schematic of AAV-CaMKIIa-hChR2-GFP/AAV-CaMKIIa-GFP virus injection and optogenetic modulation of MePV^Glu^ neurons (left panel). AAV-CaMKIIa-ChR2-GFP expression and optical fiber placement in the MePV (right panel). Scale bar = 200 μm. (**B**) Electrophysiology recordings of MePV^Glu^ neurons expressing ChR2 triggered by blue light pulses at 10 Hz. (**C**) An EEG spectrogram and EEG-EMG trace showed that 10 Hz stimulation was administered during NREM sleep. The arrowheads represent 4 and 8 Hz. The color scale represents the raw power spectral density (mV^2^). (**D**) Latencies to awaken from sleep in response to varied frequencies of visual stimulation (1 stimulation/animal). The latencies of NREM to wake were gradually reduced by the higher light stimulation at frequencies of 5 Hz, 10 Hz, 15 Hz, and 20 Hz, respectively. (**E**) Hypnograms for sleep and wakefulness. The optical stimulation of 2 hours was performed in the ChR2 group (470 nm, 10 Hz, 4 seconds/60 seconds, 2 hours). (**F**) The time spent in each state during 2 hours of light stimulation. (**G**) The wakefulness, NREM, and REM sleep duration of MePV^Glu^-ChR2 mice subjected to 2 hours of opto-stimulation (10 Hz for 4 seconds with a 56-second interval). (**H**) Open field test track plots with representative track plots (left). Time spent in the center zone and distance traveled (right). The red frame represents the central zone. (**I**) Heatmap depiction of raised plus maze testing (left) and time spent on open/closed arms (right). Mann-Whitney rank sum test for **D**. Wilcoxon signed rank test, Mann-Whitney rank sum test, or 2-way RM ANOVA with Holm-Šídák post hoc comparison for **F**. Wilcoxon signed rank test for **G** and **I**. Two-way RM ANOVA with Holm-Šídák post hoc comparison for **H**. *n* = 8 per group. All error bars are SEM. *P < 0.05, **P < 0.01, and ***P < 0.001.

After injecting the AAV-CaMKIIa-hChR2-GFP virus into the MePV, we implanted optical fibers into the MePV to produce MePV^Glu^-ChR2 mice (Figure 3A). Functional expression of ChR2 was validated using in vitro electrophysiology (Figure 3B). Optogenetic stimulation of CaMKIIa-ChR2–expressing neurons triggered the firing of action potentials of glutamatergic neurons (Figure 3B). During the light phase, we applied optical stimulation at the time of stable NREM sleep until the onset of wakefulness. The latency was defined as the optical stimulation time before the onset of wakefulness. Optogenetic stimulation of the MePV^Glu^-ChR2 neurons promoted waking from NREM sleep (Figure 3C). The latencies of NREM to wake were gradually reduced by the higher light stimulation frequencies of 5 Hz, 10 Hz, 15 Hz, and 20 Hz, respectively (Figure 3D). In addition, long-term optogenetic stimulation for 2 hours increased wakefulness and reduced NREM and REM sleep (Figure 3, E–G). Typical examples of the hypnogram, EMG track, EEG track, and EEG spectrogram for zeitgeber time 0–6 (ZT 0–ZT 6) from the MePV^Glu^-GFP mice and the MePV^Glu^-ChR2 mice are shown in Supplemental Figure 9, A and B, respectively.

No hyperactivity was caused by optogenetic stimulation of MePV^Glu^-ChR2–expressing neurons, consistent with the impact of chemogenetic activation of MePV^Glu^ neurons. The locomotion distance was not substantially different between the light-ChR2 group and the control group in the open field test (Figure 3H). The light-induced activation of MePV^Glu^-ChR2–expressing neurons increased the time spent on the closed arms and reduced the time spent on the open arms on the elevated plus maze (Figure 3I). Overall, optogenetic activation of MePV^Glu^ neurons could increase wakefulness and anxiety-like behaviors.

### MePV^Glu^ neurons promote wakefulness through the MePV^Glu^/PMCo pathway.

**Figure 4:**
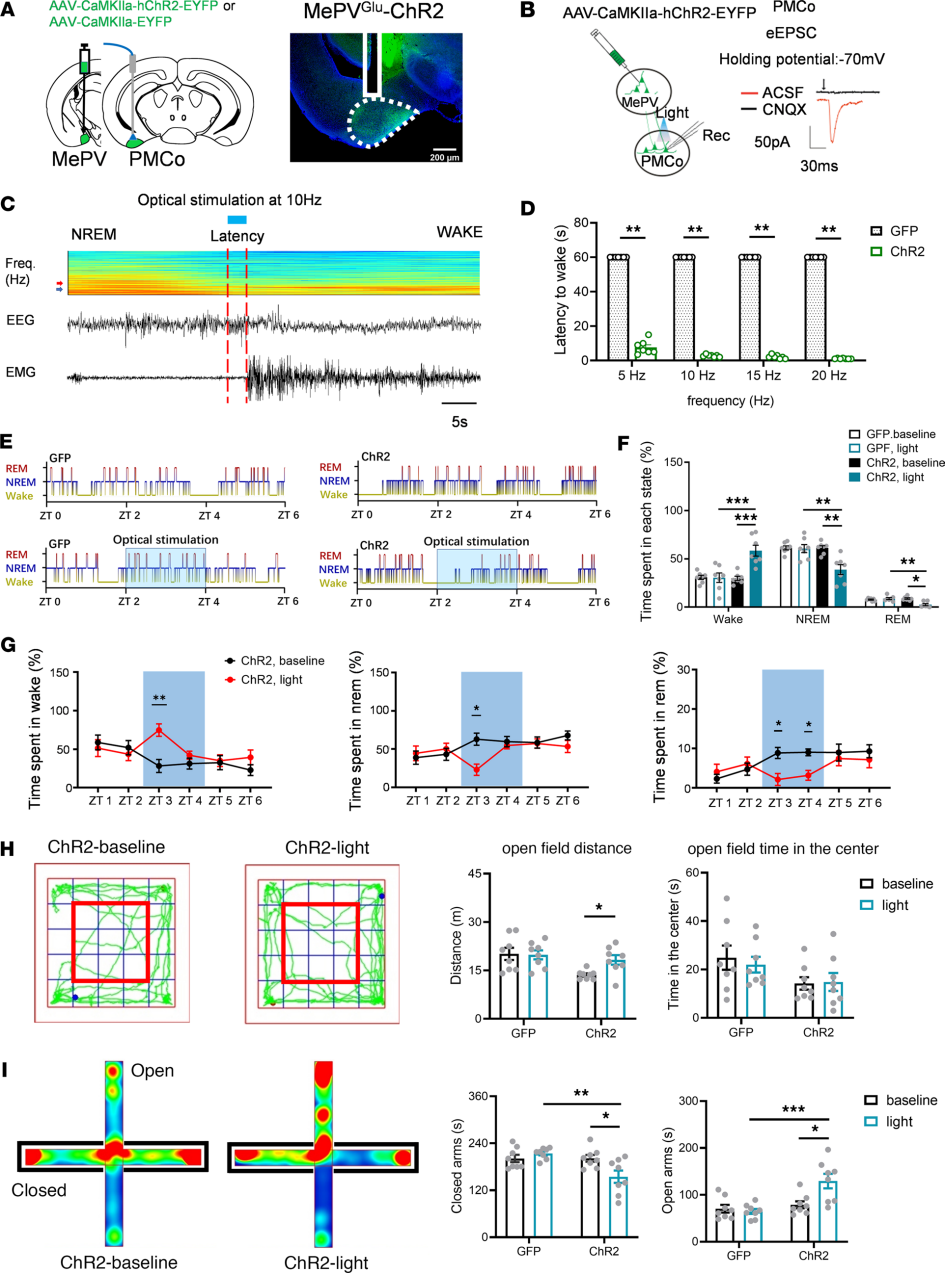
The regulation of wakefulness and anxiety-like behaviors by MePV^Glu^-PMCo circuit. (**A**) An optical fiber was implanted into the PMCo region of MePV^Glu^-ChR2 mice (left panel). The distribution of ChR2-expressing MePV glutamatergic terminals and the position of optical fiber in the PMCo (right panel). Scale bar = 200 μm. (**B**) Patch-clamp electrophysiology diagram (left panel). The eEPSC was induced by 2 Hz laser stimulation in the PMCo (right panel). (**C**) Representative EEG spectrogram and EEG-EMG recordings. During NREM sleep, 10 Hz light stimulation was administered (red arrow: 4 Hz; blue arrow: 8 Hz; Freq., frequency). The color scale represents the raw power spectral density (mV^2^). (**D**) Latencies to awaken from sleep following varied frequencies of light stimulation (once per animal, *n* = 7 per group). (**E**) A 2-hour light stimulation hypnogram during the light phase. (**F**) Time spent in each state throughout 2 hours of optogenetic stimulation (*n* = 7 per group). (**G**) The waking time, NREM time, and REM time of MePV^Glu^-PMCo ChR2 mice subjected to 2 hours of opto-stimulation (10 Hz for 4 seconds with 56-second intervals). (**H**) Representative track plots (left). The time spent in the center zone and distance traveled (right) in the open field test. The red frame represents the middle zone (*n* = 8 per group). (**I**) Elevated plus maze (EPM) heatmap representation (left) and time spent on open/closed arms (right) (*n* = 8 per group). Mann-Whitney rank sum test for **D**. Wilcoxon signed rank test, Mann-Whitney rank sum test, or 2-way RM ANOVA with Holm-Šídák post hoc comparison for **F**. Wilcoxon signed rank test, Mann-Whitney rank sum test, or paired *t* test for **G**. Two-way RM ANOVA with Holm-Šídák post hoc comparison where applicable for **H** and **I**. *P < 0.05, **P < 0.01, ***P < 0.001.

Measuring the track of viral expression and c-fos activity in MePV^Glu^-hM3Dq mice allowed targeting of the brain circuits responsible for MePV^Glu^-mediated wakefulness. Numerous brain areas showed high numbers of c-fos–positive neurons following the CNO i.p. injection. Particularly, c-fos expression was seen in the PMCo (Supplemental Figure 5). CNO infusion increased the number of c-fos–expressing neurons more than saline injection (Supplemental Figure 5). The circuit connections of MePV^Glu^ neurons in the MePV^Glu^-ChR2-GFP mice were mapped. There was a dense projection of MePV^Glu^ neurons to the PMCo, consistent with the results above (Supplemental Figure 6). Following optogenetic activation of MePV^Glu^ neuron axons ending in the PMCo, light-evoked excitatory postsynaptic currents (eEPSCs) were found in PMCo neurons using whole-cell electrophysiological recordings in brain slices (Figure 4B).

AAV-CaMKIIa-hChR2-GFP virus was injected into the MePV, and optical fibers were implanted into the PMCo to stimulate the terminals of MePV^Glu^ neurons to examine whether activation of MePV^Glu^ PMCo projections could enhance wakefulness or promote anxiety-like behaviors (Figure 4A). We found that optogenetic stimulation of MePV^Glu^ terminals in the PMCo facilitated awakening from NREM sleep. The latencies of NREM sleep to wake decreased with an increase in stimulation frequencies (Figure 4, C and D). Two-hour optogenetic stimulation enhanced alertness while decreasing REM and NREM sleep (Figure 4, E–G). Typical examples of the hypnogram, EMG track, EEG track, and EEG spectrogram for ZT 0–ZT 6 from MePV^Glu^-ChR2 mice and MePV^Glu^-GFP mice are displayed in Supplemental Figure 10, A and B.

Optogenetic activation of the MePV^Glu^ terminals in the PMCo caused hyperactivity. The locomotion distance of the light-ChR2 group was considerably more than that of the baseline-ChR2 group in the open field test (Figure 4H). The light stimulation of MePV^Glu^ terminals in the PMCo enhanced the time spent on the open arms of the elevated plus maze (Figure 4I). As a result, the MePV^Glu^/PMCo pathway is a crucial functional neural circuit that regulates wakefulness, movement, and anxiety-like behaviors. Activation of the MePV^Glu^/PMCo pathway increased wakefulness, induced anxiolytic effects, and promoted locomotion.

### MePV^Glu^ neurons promote wakefulness via the MePV^Glu^/BNST pathway.

**Figure 5:**
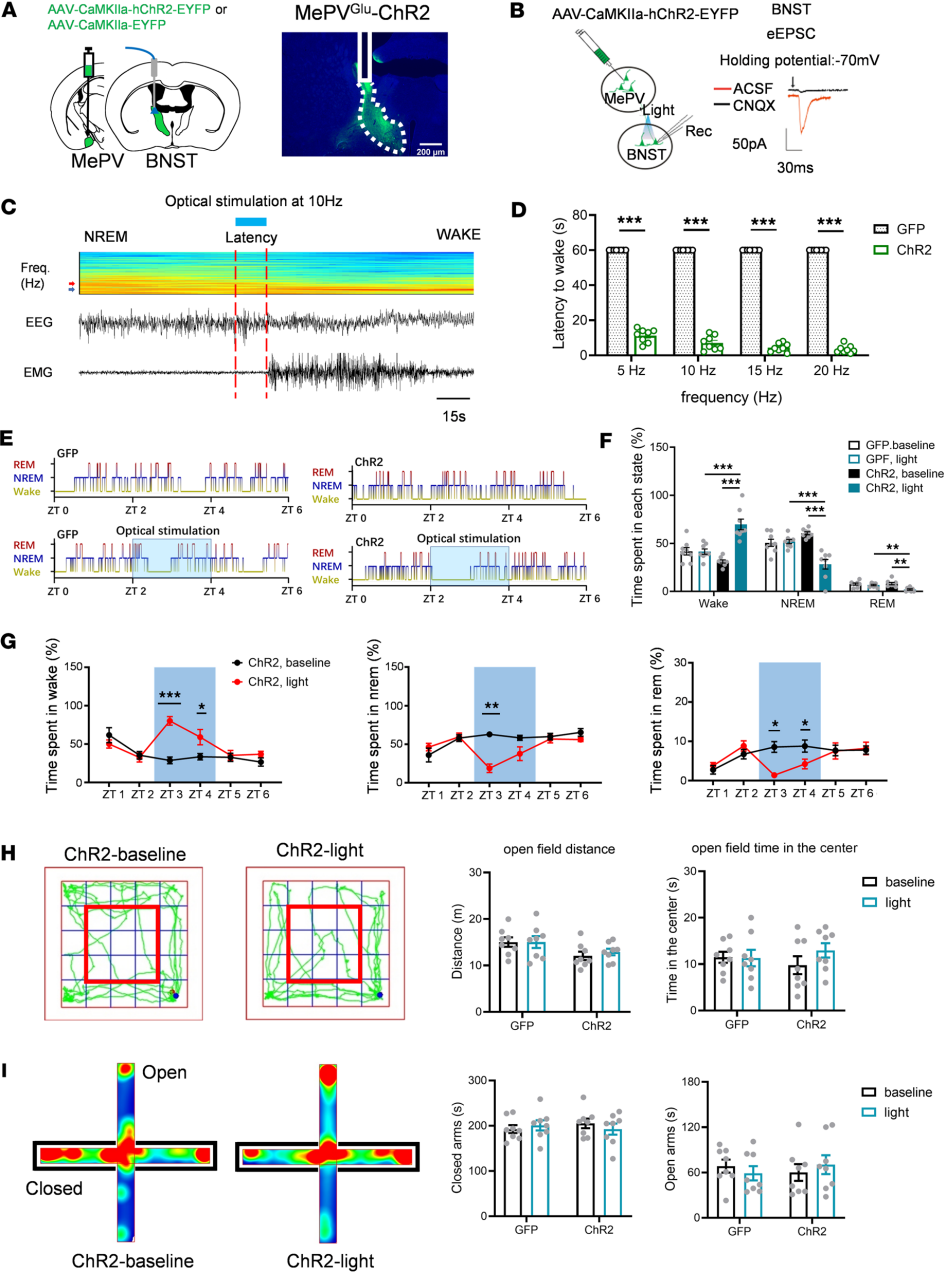
The effects of the MePV^Glu^/BNST pathway on wakefulness and anxiety-like behaviors. (**A**) An optical fiber was implanted into the BNST region of MePV^Glu^-ChR2 mice to investigate the function of MePV^Glu^-BNST projection (left). The picture depicted the distribution of ChR2-expressing MePV glutamatergic terminals in the BNST as well as the placement of optical fiber (right). Scale bar = 200 μm. (**B**) Patch-clamp electrophysiology diagram (left). In the BNST, voltage-clamp traces revealed the EPSC induced by 2 Hz laser stimulation (right). (**C**) EEG spectrogram and EEG-EMG trace showed that 10 Hz stimulation was administered during NREM sleep (red arrow: 4 Hz; blue arrow: 8 Hz). The color scale represents the raw power spectral density. The latencies of NREM to wake were shortened by the high stimulation frequencies. (**D**) Latencies to awaken from sleep following varied frequencies of optical stimulation (once/animal; GFP: 8 mice; ChR2: 8 mice). (**E**) The optical stimulation hypnograms during the light phase (470 nm, 10 Hz, 4 seconds/60 seconds, 2 hours). (**F**) Time spent in each state throughout 2 hours of light stimulation. (**G**) MePV^Glu^-BNST-ChR2 mice were subjected to 2 hours of opto-stimulation (10 Hz for 4 seconds with a 56-second interval), and the proportions of waking, NREM, and REM sleep duration were recorded. (**H**) Track plots for open field test (left). The open field test included time spent in the center zone and distance traveled (right). The red frame represents the central zone. (**I**) Heatmaps of the elevated plus maze test (left) and the time spent on the open/closed arms (right). Mann-Whitney rank sum test for **D**. Wilcoxon signed rank test, 2-way RM ANOVA with Holm-Šídák post hoc comparison for **F**–**I**. *n* = 8 per group for **D** and **F**–**I**. All error bars represent SEM. *P < 0.05, **P < 0.01, ***P < 0.001.

Dense green fluorescence nerve fiber tracts were observed in the BNST area on the MePVGlu-ChR2-GFP–labeled circuit map (Supplemental Figure 6). To test whether MePV^Glu^→BNST projections could regulate wakefulness or anxiety-like behaviors, we injected AAV-CaMKIIa-hChR2-GFP into the MePV and implanted optical fibers into the BNST to stimulate the fluorescence nerve fiber terminals (Figure 5A). Light-stimulated eEPSCs were found in BNST neurons after optogenetic activation of MePV^Glu^ neuronal terminals in the BNST using whole-cell electrophysiological recordings in brain slices (Figure 5B).

Optogenetic stimulation of the MePV^Glu^-BNST nerve fiber terminals promoted wakefulness from NREM sleep. On the other hand, the latencies of NREM to wake were shortened by the high stimulation frequencies (Figure 5, C and D). Two hours of prolonged optogenetic stimulation enhanced wakefulness and reduced REM and NREM sleep (Figure 5, E–G). Typical examples of the hypnogram, EMG track, EEG track, and EEG spectrogram for ZT 0–ZT 6 from the MePV^Glu^-GFP mice and the MePV^Glu^-ChR2 mice are shown in Supplemental Figure 11, A and B, respectively.

However, optogenetic activation of the MePV^Glu^ terminals in the BNST showed no significant impact on the elevated plus maze or open field test (Figure 5, H and I). No significant difference was observed in the locomotion distance or time spent in the center between the light-ChR2 group and the baseline-ChR2 group in the open field test (Figure 5H). The light stimulation of MePV^Glu^ terminals in the BNST had no impact on how much time was spent on the open or closed arms of the elevated plus maze (Figure 5, H and I). In all, the MePV^Glu^/BNST route was crucial for regulating wakefulness but not anxiety-like behaviors.

### MePV^Glu^ neurons increase anxiety-like behaviors via the VMH area.

**Figure 6:**
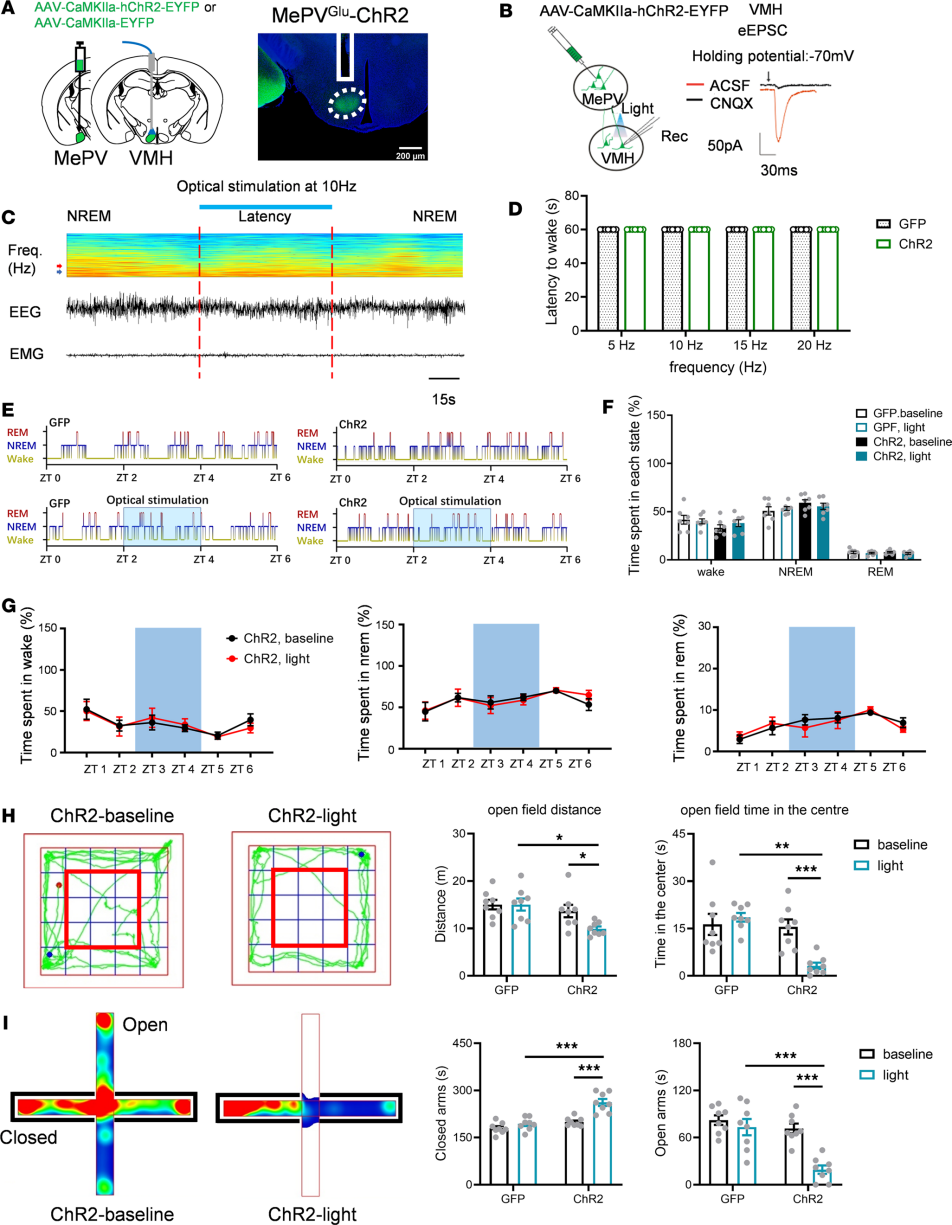
The role of the MePV^Glu^/VMH pathway on wakefulness and anxiety-like behaviors. (**A**) An optical fiber was implanted into the VMH region of MePV^Glu^-ChR2 mice to investigate the function of MePV^Glu^→VMH projection (left). The VMH picture depicted the distribution of ChR2-expressing MePV glutamatergic terminals as well as the placement of optical fiber (right). Scale bar = 200 μm. (**B**) Patch-clamp electrophysiology diagram (left). The EPSC was generated by 2 Hz laser stimulation in the VMH as seen by voltage-clamp traces (right). (**C**) An EEG spectrogram and EEG-EMG trace showed that 10 Hz stimulation was administered during NREM sleep. The arrowheads represent 4 (red) and 8 Hz (blue). The color scale represents the raw power spectral density. Fre., frequency. (**D**) Latencies to awaken from sleep following varied frequencies of optical stimulation (The stimulation was performed once per animal; GFP: 8 mice; ChR2: 8 mice). (**E**) Two-hour optogenetic stimulation hypnograms during the light phase. (**F**) Time spent in each state throughout 2 hours of light stimulation. (**G**) MePV^Glu^-VMH-ChR2 mice were subjected to 2 hours of opto-stimulation (10 Hz for 4 seconds with a 56-second interval): the proportion of waking, NREM, and REM sleep duration. (**H**) Representative open field test track plots (left). The open field test involved time spent in the center zone and distance traveled (right). The red frame represents the middle zone. (**I**) Heatmaps of the EPM test (left) and time spent on the open/closed arms (right). Mann-Whitney rank sum test for **D**. Two-way RM ANOVA with Holm-Šídák post hoc comparison for **F**–**I**. All error bars represent SEM. **D**, **F**, and **G**: *n* = 7 per group, **H** and **I**: *n* = 8 per group. *P < 0.05, **P < 0.01, ***P < 0.001.

As shown in the MePV^Glu^-ChR2-GFP–labeled circuits, some green fluorescence nerve fiber tracts were also found in the VMH area. To test whether MePV^Glu^→VMH projections could regulate wakefulness or anxiety-like behaviors, we injected the AAV-CaMKIIa-hChR2-GFP virus into the MePV and placed optical fibers into the VMH to stimulate the fluorescence nerve fiber tracts (Figure 6A). Light-evoked EPSCs were found in VMH neurons after optogenetic activation of MePV^Glu^ neuronal terminals using whole-cell path recordings in brain slices (Figure 6B).

Optogenetic stimulation of the MePV^Glu^→VMH nerve fiber terminals had no significant effect on wakefulness from NREM sleep (Figure 6, C and D). Long-term optogenetic stimulation of this pathway for 2 hours did not affect sleep and wakefulness (Figure 6, E–G). Typical examples of the hypnogram, EMG track, EEG track, and EEG spectrogram for ZT 0–ZT 6 from the MePV^Glu^-GFP mice and the MePV^Glu^-ChR2 mice are shown in Supplemental Figure 12, A and B, respectively.

Optogenetic stimulation of the MePV^Glu^→VMH fluorescence nerve fiber terminals decreased the locomotion and reduced the time spent in the center of the open field test (Figure 6H). Optogenetic activation of the MePV^Glu^/VMH pathway reduced time spent on the open arms and increased time spent on the closed arms during the elevated plus maze test (Figure 6I). Thus, the MePV^Glu^/VMH pathway was an important neuronal circuit for regulating anxiety-like behaviors but not for the modulation of sleep and wakefulness.

### No interactions between different downstream projections of MePV^Glu^ neurons.

Although we had explored the role of different downstream projections of MePV^Glu^ neurons in wakefulness and anxiety, it was still unclear whether there were reciprocal projections between downstream neurons and whether MePV^Glu^ neurons could cause back propagation resulting in cell body activation.

In order to answer the above questions, we injected AAV-CaMKIIa-hChR2-mCherry into the MePV. We also injected rAAV-CaMKIIa-GCaMP6f into the BNST, PMCo, and VMH to record the activity of glutamatergic neurons (Supplemental Figure 7) and implanted optical fibers into the BNST, PMCo, and VMH to activate the terminals of MePV neurons. After 4 weeks of recovery in the mice, we delivered blue light stimulation (10 Hz, 10 mW, 473 nm, 1-second blue light stimulation at 50-second intervals) into the terminals of MePV in the BNST. We recorded changes in the Ca^2+^ signal in the remaining 2 downstream regions, the PMCo and VMH. The same light stimulation was delivered into the PMCo and VMH, and changes in Ca^2+^ signal in these 2 downstream regions were recorded. We found that all Ca^2+^ signal was not significantly changed when the laser was delivered into the terminals of MePV neurons in the BNST, PMCo, or VMH (Supplemental Figure 7). These results suggested that there were no reciprocal projections between downstream neurons, and high-frequency stimulation of terminals might not cause back propagation resulting in cell body activation in our research.

### Inhibiting MePV^Glu^ neurons with a chemogenetic agent induces an anxiolytic effect without affecting natural sleep and wakefulness.

**Figure 7:**
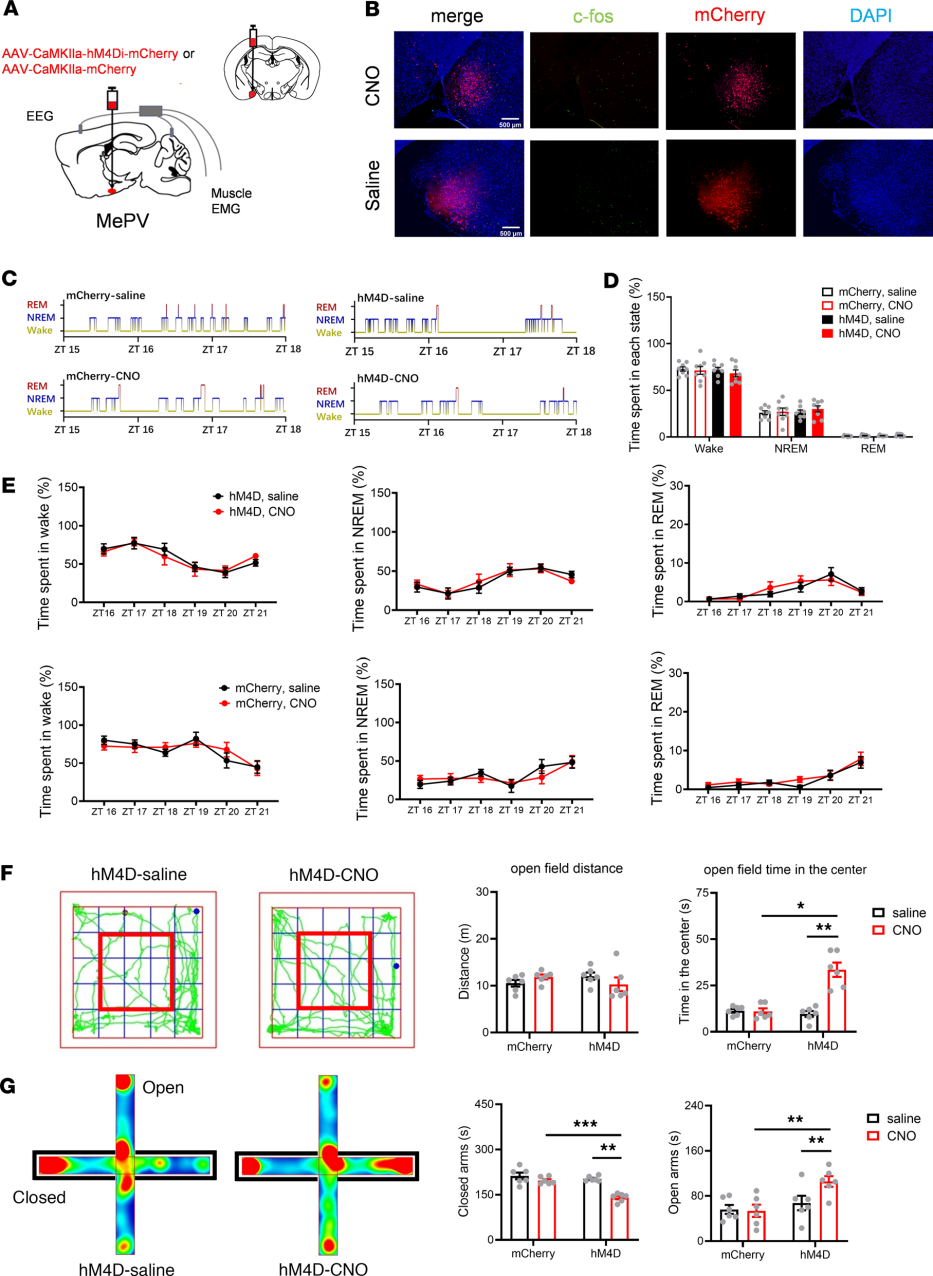
The effects of chemogenetic inhibition of MePV^Glu^ neurons on wakefulness and anxiety-like behavior. (**A**) Schematic of AAV-CaMKIIa-hM4Di-mCherry/AAV-CaMKIIa-mCherry virus injection and the EEG-EMG recordings. (**B**) Representative pictures of c-fos (green), mCherry (red), and DAPI (blue) colocalization in the MePV of hM3Dq mice treated with CNO or saline. Scale bar = 500 μm. (**C**) The 3-hour hypnograms following saline or CNO (1 mg/kg) injections into the AAV-CaMKIIa-mCherry mouse (left panel) and the AAV-CaMKIIa-hM3Dq-mCherry mouse (right panel). (**D**) Percentages of time spent in each condition for MePV-hM4D and MePV-mCherry mice 3 hours after CNO injection. (**E**) The 6-hour line charts of the AAV-CaMKIIa-hM3Dq-mCherry mouse (top panel) and the AAV-CaMKIIa-mCherry mouse (bottom panel) following saline or CNO (1 mg/kg) injections. (**F**) Representative open field test track plots (left). The open field test involved the time spent in the center zone and distance traveled (right). The red frame represents the middle zone. (**G**) Heatmaps of the EPM test (left) and time spent in the open/closed arms (right). Wilcoxon signed rank test, 2-way RM ANOVA with Holm-Šídák post hoc comparison for **D**–**G**. All error bars are SEM. **D** and **E**: *n* = 8 per group, **F** and **G**: *n* = 6 per group. *P < 0.05, **P < 0.01, ***P < 0.001.

The above results suggest that the endogenous MePV^Glu^ neurons might be related to the hyperarousal response and anxiety-like behaviors. To investigate whether inhibiting the endogenous MePV^Glu^ neurons could affect sleep or anxiety-like behaviors, we developed MePV^Glu^-hM4Di mice, in which MePV^Glu^ neurons were suppressed by injecting saline or CNO (i.p., 1 mg/kg) (Figure 7A).

We recorded and examined EEG and EMG data following saline or CNO (i.p., 1 mg/kg) injections (Figure 7A). However, sleep or wakefulness of the MePV^Glu^-hM4Di mice was unaffected by CNO injection. AAV-CaMKIIa-mCherry was injected into the MePV as an additional control for the specificity of the effect of CNO. CNO injection into these MePV^Glu^-mCherry mice had no effect on undisturbed sleep or wakefulness (Figure 7, C–E). Typical examples of the hypnogram, EMG track, EEG track, and EEG spectrogram for 3 hours from the MePV^Glu^-hM4Di mice are displayed in Supplemental Figure 13, A and B. These findings revealed that sleep and wakefulness were unaffected by the suppression of endogenous MePV^Glu^ neurons.

We next performed behavioral tests and found that inhibiting MePV^Glu^ neurons had no effect on the locomotion distance and speed in the open field test. However, the CNO-injected mice spent more time in the center in the open field test than the control group (Figure 7F). The CNO treatment could also enhance open arm time and reduce closed arm time on the elevated plus maze test. Therefore, inhibition of the MePV^Glu^ neurons reduced anxiety-like behaviors (Figure 7G). All these results suggested that the endogenous MePV^Glu^ neurons regulated anxiety-related behaviors but might not be involved in the undisturbed sleep and wakefulness of normal mice. These results indicated that MePV^Glu^ neurons could be potential targets for treating anxiety disorders.

### SD increases the activity of MePV neurons and changes the excitability of the MePV-related neuronal circuits.

**Figure 8:**
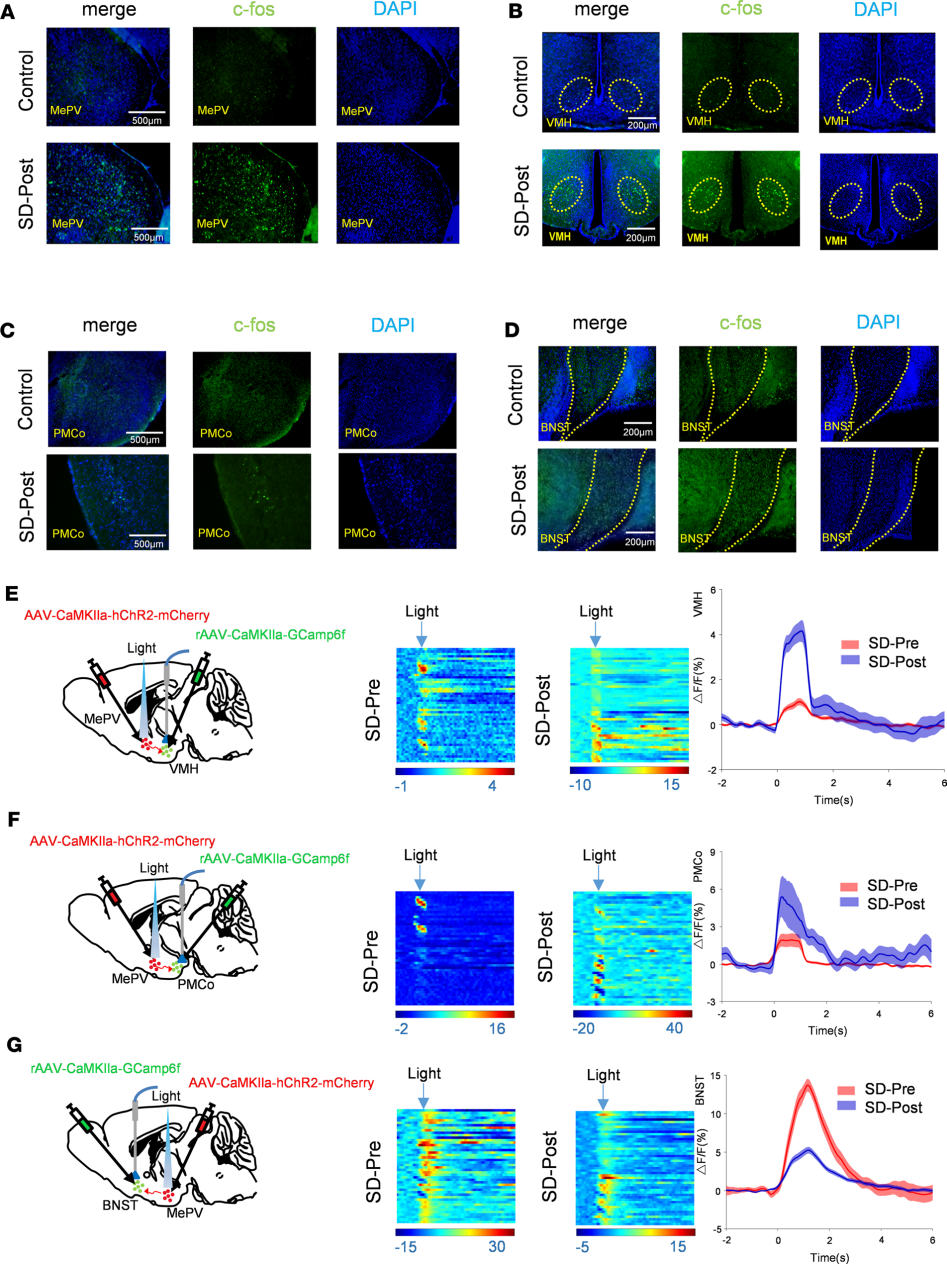
The effects of sleep deprivation on the activity of MePV neurons and the MePV-related neuronal circuits. (**A**) The expression of c-fos in the MePV of C57BL/6J mice following a 5-day sleep deprivation (SD) paradigm. Representative immunostaining images of c-fos+ cells in the MePV (**A**), VMH (**B**), PMCo (**C**), and BNST (**D**) of control and SD mice. (**E**) Left panel, experimental scheme of bilateral virus injection into the MePV and VMH. Middle panel, the heatmap for the calcium signal of VMH glutamatergic neurons before SD or after SD. The representative transitions (lights off to lights on) of the changes in color-coded fluorescence intensity before SD or after SD (*n* = 5, 50 trials). Right panel, the mean value (red trace or blue trace) represents the average responses of all the transitions (SEM: red shading or blue shading). (**F**) Left panel, experimental scheme of bilateral virus injection into the MePV and PMCo. Middle panel, color-coded fluorescence intensity changes of the representative shifts from the lights-off phase to the light-on phase before SD or after SD (*n* = 5, 45 trials). Right panel, the mean value (red trace or blue trace) represents the average responses of all the transitions (SEM: red shading or blue shading). (**G**) Left panel, experimental scheme of bilateral virus injection into the MePV and BNST. Middle panel, a shift in color-coded fluorescence intensity illustrates the representative transition from the lights-off phase to the light-on phase before SD or after SD (*n* = 5, 47 trials). Right panel, the mean value (red trace or blue trace) represents the average responses of all the transitions (SEM: red shading or blue shading). Data are presented as mean ± SEM.

The above results suggest that though endogenous MePV^Glu^ neurons might not be involved in undisturbed sleep-wake transitions, the endogenous MePV^Glu^ neurons might be related to sleep disruption and anxiety disorders. As we know, chronic passive sleep disturbance could worsen anxiety disorders and change the structure of sleep, but little research has been done on the mechanisms. We first tested the effect of chronic sleep deprivation on the activity of MePV neurons and MePV-related neuronal circuits using c-fos immunohistochemical staining. Numerous brain areas showed high numbers of c-fos–positive neurons following sleep deprivation. Notably, sleep deprivation substantially increased the number of c-fos–expressing neurons in the MePV, VMH, and PMCo areas (Figure 8, A–C, and Supplemental Figure 14). However, sleep deprivation did not significantly affect the number of c-fos–expressing neurons in the BNST area compared with the control group (Figure 8D and Supplemental Figure 14).

To test whether chronic SD affected the real-time activity of MePV neurons and neuronal circuits, we injected AAV-CaMKIIa-hChR2-mCherry into the MePV and implanted optical fibers into the MePV to activate the MePV neurons. We also injected rAAV-CaMKIIa-GCaMP6f into the BNST, PMCo, and VMH to record the activity of glutamatergic neurons (Figure 8). We found that the level of Ca^2+^ signal in the BNST, PMCo, and VMH all increased when the laser was delivered into the MePV (Figure 8, E–G). Compared with the control group, SD markedly increased the laser-evoked Ca^2+^ signal of glutamatergic neurons in the PMCo and VMH groups (Figure 8, E and F). But the SD markedly decreased the laser-evoked Ca^2+^ signal of glutamatergic neurons in the BNST group (Figure 8G). All these results suggested that chronic SD increased the activity of MePV^Glu^ neurons and changed the excitability of the downstream circuits. We then hypothesized that MePV^Glu^ neurons might participate in chronic SD-induced abnormal changes in anxiety and sleep.

### Inhibiting MePV^Glu^ neurons with a chemogenetic agent decreases wakefulness and induces an anxiolytic effect on the mice treated with SD.

**Figure 9:**
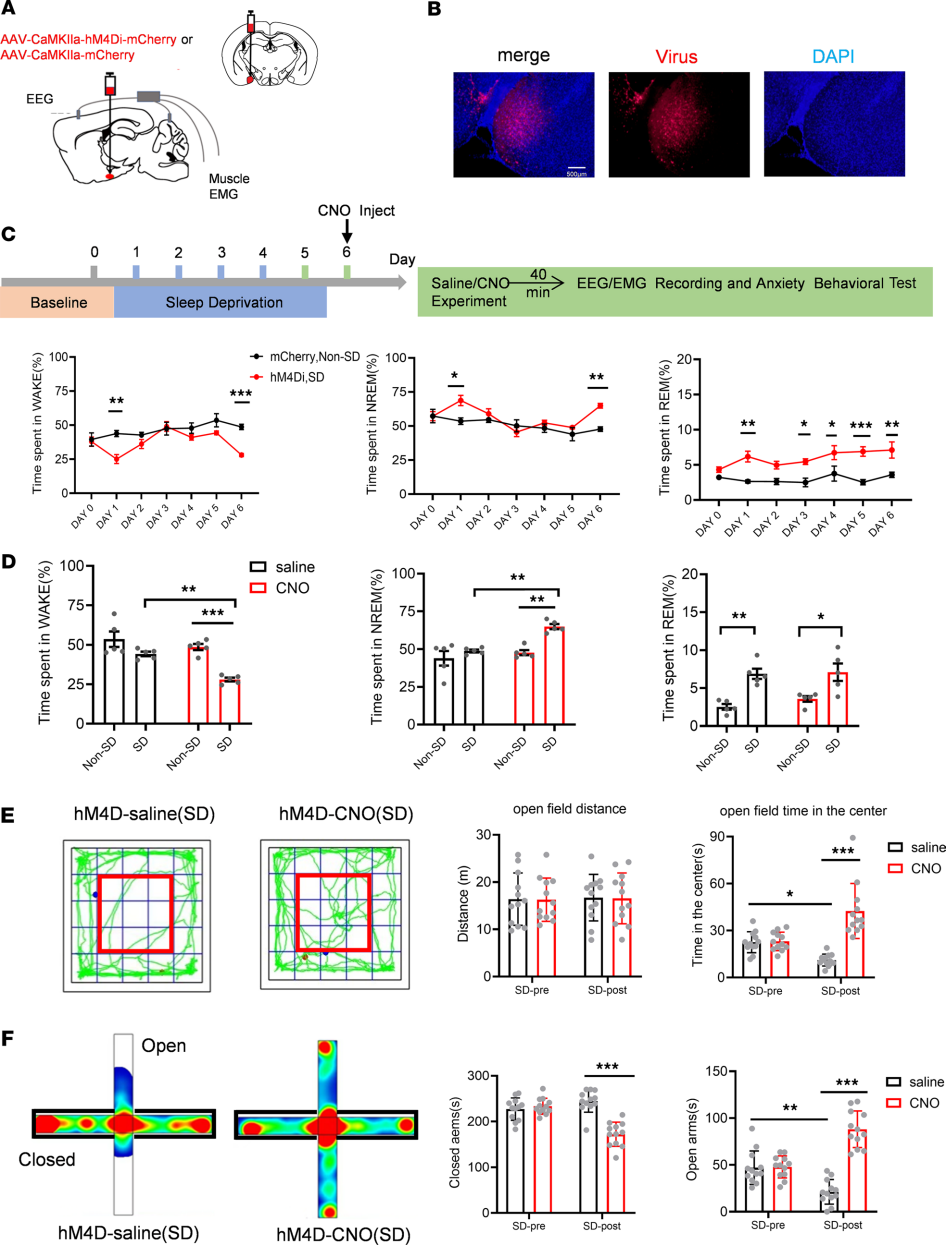
Inhibiting MePV^Glu^ neurons with a chemogenetic agent decreases wakefulness and induces an anxiolytic effect on the mice treated with SD. (**A**) Schematic of AAV-CaMKIIa-hM4Di-mCherry/AAV-CaMKIIa-mCherry virus injection and the EEG-EMG recordings. (**B**) Representative pictures of virus (red), DAPI (blue), and colocalization in the MePV of hM3Dq mice. (**C**) Upper panel, schematic diagram of the experimental process. Mice were subjected to SD from day 1 to day 5. On day 6, mice were given saline or CNO separately. We recorded and examined EEG and EMG data for 6 hours immediately after the SD or the CNO treatment (*n* = 5 mice). Lower panel, the statistical analysis results of wakefulness time, NREM sleep time, and REM sleep time for 6 days. (**D**) The statistical analysis results of wakefulness time, NREM sleep time, and REM sleep time on day 6 (*n* = 5 mice). (**E**) Representative traces of open field test (left). The red frame represents the middle zone. The statistical analysis results of open field tests involved distance traveled and the time spent in the center zone (right) (mCherry: *n* = 12 mice; hM4Di: *n* = 12 mice). (**F**) Heatmaps of the EPM test (left) and time spent on the EPM open/closed arms (right). Statistical significance was determined using the 2-way RM ANOVA. Data are presented as mean ± SEM. *P < 0.05, **P < 0.01, ***P < 0.001.

To investigate whether chronic SD could affect sleep and anxiety-like behaviors, or whether inhibiting the endogenous MePV^Glu^ neurons could reverse the effect of SD, we developed MePV^Glu^-hM4Di mice, of which MePV^Glu^ neurons were suppressed by injecting saline or CNO (i.p., 1 mg/kg) (Figure 9, A and B). We subjected the mice to 5 days of SD and recorded and examined EEG and EMG data for 6 hours immediately after the SD every day (Figure 9C). We found that 1 day of SD significantly decreased wakefulness and increased NREM sleep and REM sleep (Figure 9C). Because the mice were forced to stay awake during the SD, the increased sleep after the SD on day 1 was reparative. But from the second day on, the wakefulness increased and NREM sleep decreased (Figure 9C), which suggested that the mice experienced a significant reduction in reparative sleep, and had a relative increase in total wakefulness and a relative decrease in NREM sleep duration. On the sixth day after the SD, we injected CNO (i.p., 1 mg/kg) to inhibit the MePV^Glu^ neurons, and we found that the CNO treatment significantly decreased the wakefulness time of the SD group compared with the non-SD group (Figure 9, C and D). The NREM sleep of the SD group was significantly increased by the CNO treatment compared with that of the non-SD group (Figure 9, C and D). Five days of SD increased the REM sleep (Figure 9, C and D), but the CNO treatment did not affect the REM sleep time in both the non-SD group and the SD group (Figure 9, C and D).

To study the effects of SD on the activity of MePV^Glu^ neurons, we recorded the Ca^2+^ signals of MePV^Glu^ neurons from NREM to arousal transitions after 1 day or 5 days of SD (Supplemental Figure 2, E and F). After the first day of SD, the Ca^2+^ signal of MePV^Glu^ neurons was decreased during NREM-wake transitions (Supplemental Figure 2E). However, 5 days after SD, the Ca^2+^ signal of MePV^Glu^ neurons was increased during NREM-wake transitions (Supplemental Figure 2F). All these results suggested that the decreased activity of MePV^Glu^ neurons during NREM-wake transitions caused a rebound of NREM sleep on day 1, and SD-mediated increased activity of MePV^Glu^ neurons during NREM-wake transitions might promote wakefulness after chronic SD.

We next performed behavioral tests and found that inhibiting MePV^Glu^ neurons of the SD group did not affect the locomotion in the open field test compared with the saline group. However, the CNO-injected mice spent more time in the center in the open field test than the SD-saline group (Figure 9E). The CNO treatment after the SD could also increase open arm time and decrease closed arm time in the elevated plus maze test compared with the SD-saline group (Figure 9F). Therefore, inhibition of the MePV^Glu^ neurons reduced anxiety-like behaviors after chronic SD. All these results suggested that the endogenous MePV^Glu^ neurons might be involved in SD-mediated obsessive anxiety-like behavior and sleep change. Inhibition of the MePV^Glu^ neurons reduced anxiety-like behaviors after SD and promoted restorative sleep. These results indicated that MePV^Glu^ neurons could be potential targets for treating anxiety disorders and insomnia.

## Discussion

Exposure to threatening situations increases alertness and arousal response to the conditions. On the other hand, overactivation of the limbic system that regulates wakefulness could cause hyperarousal (22). Sleep disruption is frequently present with mental health disorders, such as anxiety disorders, posttraumatic stress disorder, and depression. Elucidating the neural circuits that regulate physiological arousal in response to threats is essential for understanding maladaptive emotional states and stress-evoked sleep changes. We found that MePV^Glu^ neurons were activated by threat-evoked awakening from NREM sleep. Excitation of MePV^Glu^ neurons promoted wakefulness and increased anxiety-like behaviors. Different neural circuits of MePV^Glu^ neurons played different roles in regulating anxiety-like behaviors and sleep-wakefulness. MePV^Glu^ neurons promoted wakefulness through the MePV^Glu^/PMCo and MePV^Glu^/BNST pathways but not through the MePV^Glu^/VMH pathway. In contrast, MePV^Glu^ neurons promoted anxiety-like behaviors mainly through the MePV^Glu^/VMH pathway. Our studies suggested that MePV^Glu^ neurons play a role in regulating anxiety-like behaviors and arousal responses to threatening stimuli. Overactivation of MePV^Glu^ neurons could cause hyperarousal response and obsessive anxiety-like behavior. Chemogenetic inhibition of MePV^Glu^ neurons produced an anxiolytic effect without affecting natural sleep-wake transitions.

After studying the neural circuits that control arousal for threat detection, we explored the adaptive responses to chronic SD. Chronic passive SD increased anxiety-like behaviors and changed the structure of sleep, but the underlying mechanisms were barely known. We then explored the effect of chronic SD on the activity of MePV neurons and downstream neuronal circuits using c-fos immunohistochemical staining and the fiber photometry. We found that 5 days of SD activated the MePV^Glu^ neurons and increased MePV^Glu^ neuron–induced responses of PMCo and VMH glutamatergic neurons. However, MePV^Glu^ neuron–induced responses of BNST glutamatergic neurons were decreased by SD. We next found that 1-day SD significantly decreased wakefulness and increased NREM sleep and REM sleep (Figure 9C). Because the mice were forced to stay awake during the SD, the increased sleep after the SD on day 1 was reparative. But from the second day on, the wakefulness increased and NREM sleep decreased (Figure 9C), which suggested that the mice experienced a significant reduction in reparative sleep, and had a relative increase in total wakefulness and a relative decrease in NREM sleep duration. We next found that the inhibition of the MePV^Glu^ neurons reduced anxiety-like behaviors, decreased wakefulness, and promoted NREM sleep after the SD. The endogenous MePV^Glu^ neurons might inhibit restorative sleep after chronic SD. These results indicated that MePV^Glu^ neurons could be targets for treating anxiety disorders and sleep disturbance. Although excitation of MePV^Glu^ neurons could decrease REM sleep in normal mice, inhibition of MePV^Glu^ neurons had no effect on REM sleep, with or without the SD. This phenomenon is due to the complexity and diversity of neural circuit functions. Further investigations should be made to explain it.

Studies have revealed lower alertness to predator odor during REM sleep than in NREM sleep in rats (23). Previous studies have also demonstrated that the neuronal circuit comprising corticotropin-releasing factor neurons in the medial subthalamic nucleus regulated awakening from REM sleep by quickly integrating olfactory and visual signals (23). However, some other studies have revealed that acute optogenetic stimulation of GABAergic neurons in the BNST during NREM sleep causes an immediate transition to wakefulness. In contrast, stimulation during REM sleep has no impact on the sleep-wakefulness states in male mice (24). We speculate that regardless of whether the rapid awakening of animals depends on REM or NREM, it is closely related to the functions of different brain regions and circuits and the stimulus encountered by animals. In our experiments, sleep-wake transitions were evoked by the loud noise of opening the soundproof door, which mimicked intrusion during sleeping. We found that MePV^Glu^ neurons were selectively active in noise-evoked awakening and from NREM sleep to REM sleep and changed little during natural NREM-to-wake transitions. Our data are consistent with previous experimental evidence that the human amygdala was activated during REM (25, 26). Some other studies have shown that REM is necessary for fear memory consolidation (27). Consistent with the result of in vivo calcium imaging recording, no effects were observed on sleep and wake time in the chemogenetic inhibition of endogenous MePV^Glu^ neurons. However, chemogenetic excitation of MePV^Glu^ neurons could promote wakefulness. These results suggested that endogenous MePV^Glu^ neurons might not affect wakefulness in normal states. MePV^Glu^ neurons only participated in responding to threats during sleep, and overexcited MePV^Glu^ neurons could lead to insomnia disorders. We also found that inhibiting the MePV^Glu^ neurons reduced anxiety-like behaviors, decreased wakefulness, and promoted NREM sleep after the 5 days of SD. We supposed that there might be a threshold for these neurons to promote wakefulness. The activity of MePV^Glu^ neurons was below the threshold in the normal mice, and inhibiting these neurons did not cause changes in wakefulness. However, acute threats or SD could increase neuronal activity exceeding the threshold range and thus promote wakefulness. Consistent with this inference, we found that the Ca^2+^ signal of MePV^Glu^ neurons was increased during NREM-to-wake transitions after 5 days of SD (Supplemental Figure 2F). This could also explain why chemogenetic inhibition of endogenous MePV^Glu^ neurons could promote NREM sleep after SD but could not affect sleep in normal mice.

According to the results of the elevated plus maze test and open field test, chemogenetic excitation of MePV^Glu^ neurons enhanced anxiety-like behaviors, whereas chemogenetic inhibition of MePV^Glu^ neurons could alleviate anxiety-like behaviors. The results of fiber photometry during the elevated plus maze showed high activity of MePV^Glu^ neurons when mice tried to explore the open arms, which decreased when mice entered the closed arms. Similarly, a previous experiment showed an association between anxiety-like behaviors of rats and c-fos expression in MePV (13). Thus, we speculate that MePV^Glu^ neurons influence anxiety-related behaviors. Previous studies have shown that the PMCo might regulate male reproductive behavior, social odor, and primary olfactory processing (18, 24). The PMCo has feedback circuit connectivity with the accessory olfactory bulb and dense bilateral connectivity with the Me posterior (18, 28). However, the functions of PMCo on wakefulness-sleep or anxiety are few. To identify the brain circuits that generate MePV^Glu^/PMCo pathway–mediated wakefulness, we measured c-fos activity in MePV^Glu^-hM3Dq mice. Many c-fos–positive cells were identified in multiple brain regions, especially in the PMCo. We thus investigated the function of the PMCo and found that the MePV^Glu^/PMCo pathway was an important “gate” for controlling wakefulness but not anxiety-like behaviors. Herein, we found that activation of the MePV^Glu^/PMCo pathway increased the time spent on open arms in the elevated plus maze test and promoted locomotion in the open field test. Thus, stimulation of the MePV^Glu^ terminals in the PMCo could enhance motor ability. Our studies provided evidence for the study of PMCo in sleep-wake research and provided groundbreaking direction for subsequent research.

Previous studies have suggested that regulation of anxiety-like behaviors requires the function of the BNST, whereas excitation of the BNST results in high anxiety-like behaviors (29, 30). Kim et al. identified that BNST coordinated the modulation of diverse anxiety features in which distinct subregions exert opposite effects in modulating anxiety (31). The BNST GABAergic system played a role in sleep-wakefulness control, and reward-promoting cholecystokinin-BNST neurons received their dense inputs from the Me, which provided an essential mechanism underlying the emotional arousal regulation and the pathophysiology of insomnia (24, 32, 33). We found that excitation of MePV^Glu^ neuronal terminals projecting to BNST promotes wakefulness. However, the MePV^Glu^/BNST pathway did not participate in the regulation of anxiety-like behaviors. We also found that chronic SD could decrease the responses of BNST glutamatergic neurons induced by optogenetic stimulation of MePV^Glu^ neurons. Therefore, the terminals of MePV^Glu^ neurons might regulate wakefulness mainly by controlling glutamatergic neurons of the BNST but not GABAergic neurons. Otherwise, more complicated microneuronal circuits in the BNST might also play a role in controlling GABAergic neurons. However, there is a need for further investigations to validate these findings.

The GABAergic neural circuits and astrocytes of the VMH regulate anxiety and metabolism (34, 35). Several studies in related fields have indicated that the Me and the VMH are specific subregions involved in fear and anxiety caused by predator odor (36, 37). Our data on MePV^Glu^/VMH are consistent with previous studies. In our experiments, excitation of MePV^Glu^ neuronal terminals projecting to VMH increased anxiety-like behaviors with no effect on sleep-wakefulness. It means that neural circuits of MePV^Glu^/VMH may participate in mouse anxiety caused by predator odor. There is a need for additional studies to investigate the mechanism underlying neural circuits.

The changes in wake time induced by optogenetic stimulation were more pronounced than changes in the second hour, as seen in Figure 3E, Figure 4E, and Figure 5E. One explanation is that the stimulation might produce a short-term effect on arousal that could be quickly compensated for by sleep pressure. Another possible reason is the fatigue of neurons after long-term excitation. Even if this hypothesis were true, the neuronal fatigue was short-term. During the experiment, we found that when these neurons were activated again the next day, they could still quickly promote arousal. Further investigations should be done on the impact of different durations of optogenetic stimulation on neuronal excitability. We will also conduct in-depth explorations of its mechanism in future work.

We found that the Ca^2+^ signal increased dramatically from NREM to REM sleep (Figure 1E) but dropped from REM to natural wake (Figure 1F). These results suggested that MePV^Glu^ neurons might be active during REM sleep. Though activation of MePV^Glu^ neurons could decrease REM sleep (Figure 2D and Figure 3F), inhibition of MePV^Glu^ neurons did not affect NREM and REM sleep (Figure 7). These inconsistent results illustrated the complexity of neuronal activity in nature. The mechanism by which MePV^Glu^ neurons regulate REM sleep might be as follows: There was a threshold for these neurons to promote the REM sleep transition. Below the threshold activity, although the transition to REM sleep might cause an increase in the activity of these neurons, it was only a concomitant activity and might not participate in determining the REM sleep transition. Therefore, inhibiting these neurons did not cause changes in REM sleep. However, if these neurons were highly activated by the optogenetic stimulation, the increased neuronal activity exceeding the threshold range would cause a decrease in REM sleep. Another possible reason was that a reduction in NREM sleep mediated by optogenetic stimulation would also lead to a reduction in the transitions from NREM to REM.

There might be different subclasses of MePV^Glu^ neurons responsible for different functions, because 5 days of SD could lead to a decrease in NREM sleep, an increase in REM sleep, and an increase in anxiety-like behaviors. Inhibiting these neurons reduced anxiety-like behaviors and promoted NREM sleep but did not affect the changes in REM sleep after SD. These results suggested that the REM-related neurons might be a different subclass than those that regulate NREM sleep and anxiety-like behaviors. MePV^Glu^ neurons involved in REM sleep regulation might have other compensatory neural projections or even more complex neural circuit mechanisms, requiring further study. This also pointed out the direction for our subsequent research.

In summary, our findings suggest that the endogenous MePV^Glu^ neurons play a role in anxiety-related behaviors, but activation of MePV^Glu^ neurons could cause hyperarousal response and obsessive anxiety-like behavior. Our study might provide critical underlying mechanisms for insomnia and anxiety disorders. In addition, this study provides a perspective on treating anxiety disorders.

## Methods

### Sex as a biological variable.

Male C57BL/6J wild-type mice were used for the experiments. Previous findings showed that the high hormone phase of proestrus in female mice increased wakefulness and decreased both NREM and REM sleep compared with other estrous phases and in males (38).

### Animals.

All animal experiments were conducted in strict adherence to guidelines by the National Research Council *Guide for the Care and Use of Laboratory Animals* (National Academies Press, 2011). C57BL/6J (Beijing Vital River Laboratory Animal Technology Co., Ltd.) wild-type mice (male, weighing 25–30 g, and aged 8–12 weeks) were randomly chosen and used for the experiments. All mice were housed under a controlled environment, at a constant temperature of 22 ± 2°C, 50%–60% humidity, and a 12-hour light/12-hour dark cycle that began at ZT 0. The animals were allowed unrestricted access to food and water. All behavioral tests were performed during the light cycle between 9000 hours and 1700 hours. Both the number of mice used and suffering were kept to a minimum.

### Virus injection.

The projection targets were set as follows: MePV: anteroposterior (AP): –1.58 mm, mediolateral (ML): 2.02 mm, dorsoventral (DV): –5.50 mm; BNST: AP = –0.25 mm, ML = ±0.75 mm, DV = –3.80 mm; VMH: AP = –1.45 mm, ML = ±0.55 mm, DV = –5.60 mm; PMCo: AP = –2.80 mm, ML = ±2.95 mm, DV = –5.50 mm. The virus was injected using a micropipette with a 20 µm aperture fitted with the Auto-Nanoliter Injector (Harvard Apparatus) at a rate of 10 nL/min. After microinjection, the micropipette was placed and held for 10 minutes to ensure vector diffusion, then gently removed. Detailed information is provided in Supplemental Methods.

### EEG-EMG recordings and analysis.

Detailed information is provided in Supplemental Methods.

### Fiber photometry.

Preoperative preparations for implantation of the optic fibers were performed as described above, 2 weeks following virus injection. The mice were implanted with optical fibers with a 200 µm diameter and a numerical aperture (NA) of 0.37 (Inper Tech). Briefly, the fiber was first carefully dropped over the MePV (bregma coordinates: AP = 1.58 mm; ML = +2.02 mm; DV = –5.35 mm). Other fiber positions were set as follows: BNST: AP = –0.25 mm, ML = ±0.75 mm, DV = –3.80 mm; VMH: AP = –1.45 mm, ML = ±0.55 mm, DV = –5.60 mm; PMCo: AP = –2.80 mm, ML = ±2.95 mm, DV = –5.50 mm. Then the mice were implanted with EEG electrodes as described above. Next, the fiber was attached to the surface of the skull using a small coating of Nissin Super-Bond C&B dental cement, and then a second coat of regular dental cement was used to securely bind the fiber. The mice were kept separately after surgery to ensure proper recovery.

For fiber photometry tests, detailed information is provided in Supplemental Methods. We primarily recorded the Ca^2+^ dynamics at 2 event-related time intervals during the elevated plus maze tests for the Ca^2+^ signal analysis: (A) when the mice reached the open arms and (B) when they made their way back to the closed arms. We also captured the Ca^2+^ signal 10 seconds before and 10 seconds after the events.

We primarily recorded Ca^2+^ dynamics at 6 event-related periods during the wake-sleep experiments as follows: (A) NREM – threat-evoked awakening, (B) NREM-REM, (C) REM-natural wake, (D) NREM-natural wake, (E) REM-microarousal-NREM, and (F) NREM-microarousal-NREM. The Ca^2+^ signal was captured 30 seconds before until 30 seconds after the events. For threat-evoked awakening, sleep-wake transitions were elicited by the jarring sound of opening the soundproof door to the recording chamber, which mimicked an intruder’s stimulus while the mice were asleep.

### Fiber ferrule implantation.

Implantation of the fiber ferrule was performed 2 weeks after virus injection using the procedure described above. Summarily, mice were implanted with an optical fiber ferrule (diameter: 200 µm, NA: 0.37, Inper Tech) above the MePV^Glu^ neurons for optical stimulation (same coordinates as fiber photometry). To stimulate MePV output projections, fiber ferrules were implanted into the VMH nucleus, BNST, and PMCo. Fiber positions were set as follows: MePV: AP = 1.58 mm; ML = +2.02 mm; DV = –5.0 mm; BNST: AP = –0.25 mm, ML = ±0.75 mm, DV = –3.30 mm; VMH: AP = –1.45 mm, ML = ±0.55 mm, DV = –5.10 mm; PMCo: AP = –2.80 mm, ML = ±2.95 mm, DV = –5.0 mm.

### Optical stimulation.

Mice were hooked up to optical cables, 5 days after implantation of EEG-EMG electrodes, then allowed at least 2 days to acclimatize to the recording box. A waveform generator was used to control lasers that emit blue light at 473 nm (Thinker Tech). Next, a 473 nm laser’s output power at the fiber’s tip was measured between 5 and 8 mW using an optical power meter (PM100D, Thorlabs). During NREM or REM sleep, random pulses of 473 nm light of 10 ms width at 5 to 20 Hz were administered to optogenetically excite MePV^Glu^ neurons and MePV projections. The EEG-EMG signal was visually monitored in real time, and optical stimulation was applied after the onset of stable NREM sleep (~5 minutes from onset) until awake or until 60 seconds passed. If the mouse did not wake up within 60 seconds, the latency period was defined as 60 seconds. We used the sleeping posture (curled up, still, and eyes closed) and the EEG-EMG of the mouse to determine whether the mouse had entered sleep. The NREM-REM transitions occurred during sleep. About 5 minutes after the mouse entered sleep, the new onset of NREM sleep was chosen, and the optical stimulus was delivered. For extended stimulation, from ZT 2 to ZT 4, 4 seconds in every minute and 10 ms width pulses at 10 Hz were used. Blue light stimulation (wavelength: 470 nm; frequency: 10 Hz; width: 10 ms; power: 5–8 mW) was administered through the optical cannula during the experimental stage of the elevated plus maze or open field test. All optogenetic experiments were performed between ZT 2 and ZT 10.

### Elevated plus maze.

Mice were allowed to acclimatize to the testing space for 30 minutes and then put on the raised plus maze. The elevated plus maze comprised 3 sections as follows: 2 opposed closed arms (50 × 10 cm each) with 40 cm tall opaque walls, 2 opposite open arms (50 × 10 cm each), and a middle section (10 × 10 cm). Additionally, the experimental equipment was raised 50 cm above the ground. The animals were initially positioned in the labyrinth facing the closed arm, and then the time spent on open and closed arms was recorded over a 5-minute period. After the trial, the experimental box was cleaned of mouse waste and treated with ethanol on cotton to get rid of the scent of the preceding mouse. Less exploration on open arms is linked to anxiety-related behaviors (39, 40).

### Open field test.

Mice were given at least 30 minutes to get used to the testing room, positioned in the corner of the opening experimental box (50 cm × 50 cm × 40 cm), and watched for 5 minutes. Average movement speeds (mm/s) and total lengths traveled (m) throughout the center period were measured. After the trial, the experiment box was cleaned of mouse waste and treated with cotton soaked in ethanol to remove the scent of the preceding mouse. Less center area exploration is linked to anxiety-related behaviors.

### Electrophysiological analysis.

The patch-clamp recording was performed as previously described (41, 42). Detailed information is provided in Supplemental Methods.

### Histology.

Immunohistochemistry was performed as described previously (43, 44). Detailed information is provided in Supplemental Methods.

### SD.

SD was achieved from day 1 to day 5 using an automated SD system as described previously (45, 46) Detailed information is provided in Supplemental Methods.

### Statistics.

Data were presented as mean ± SEM. Sample sizes were calculated based on past publications using optogenetic and chemogenetic approaches to investigate the sleep-wake circuitry (43, 44). The normality of each data set was initially assessed using the Shapiro-Wilk test, and those that conformed to a normal distribution were subjected to parametric analyses. We used paired or unpaired 2-tailed *t* tests to compare the means between the 2 groups. Non-normally distributed data sets were analyzed using the Mann-Whitney rank sum or the Wilcoxon signed rank tests. Comparisons across 3 or more groups were performed using 1-way ANOVA test or 2-way ANOVA test, followed by Holm-Šídák post hoc test. All statistical analyses were performed using SPSS 26.0, GraphPad Prism 9.0, or Matlab R2018a where necessary. Statistical significance was set at *P* < 0.05.

### Study approval.

All experiments were approved by the Institute of Animal Care Committee at Wuhan University’s Zhongnan Hospital in Wuhan, Hubei Province, China.

### Data availability.

All data needed to evaluate the conclusions in the paper are present in the paper and/or the supplement. Values for all data points shown in graphs and behind any reported means are available in the Supporting Data Values.

## Author contributions

HX, LB, PC, and Ying Li conceived the study; YD, Ying Li, and YZ developed methodology; YD, Ying Li, YZ, DX, XZ, Yue Li, Yidan Li, MC, YW, JZ, LW, and YC investigated; Ying Li and LB wrote the original draft; HX, LB, and PC reviewed and edited the manuscript; HX and LB acquired funding; HX and LB provided resources; and HX, LB, and PC supervised.

## Supplementary Material

Supplemental data

Supporting data values

## Version 1 06/06/2024

In-Press Preview

## Version 2 07/22/2024

Electronic publication
