# Supplementary material for: Distinct glutamatergic projections of the posteroventral medial amygdala play different roles in arousal and anxiety
Source: JCI Insight. 2024 Jun 6;9(14):e176329. doi: 10.1172/jci.insight.176329 (PMC11383360; doi:10.1172/jci.insight.176329)
Supplement: Supplemental data [file jciinsight-9-176329-s062.pdf]

Supplemental Experimental Procedures:

Virus injection and behavioral studies

Mice (8–12 weeks old) were first anaesthetized via intraperitoneal injection (i.p.) of sodium pentobarbital (50 mg/kg) (RWD Life Technology Co. Ltd., China), and positioned on a stereotaxic device. Ophthalmic ointment was administered to the mouse's eyes to stop corneal dryness and eye harm. Next, the scalp was shaved and cleaned with iodine and 70% alcohol, and then a longitudinal incision was made along the midline to reveal the skull. With the aid of the microscope, tiny holes were bored above the MePV (AP: -1.58 mm, ML: 2.02 mm, DV: -5.50 mm) for viral injections (RWD Life Technology Co. Ltd., China). Other projection targets were set as follows: BNST: AP = -0.25mm, ML = ±0.75mm, DV = -3.80 mm; VMH: AP = -1.45mm, ML = ±0.55 mm, DV = -5.60 mm; PMCo: AP = -2.80 mm, ML = ±2.95 mm, DV = -5.50 mm. The virus was injected using a micropipette with a 20 m aperture, fitted with the Auto-Nanoliter Injector (Harvard) at a rate of 10 nl/min. After microinjection, the micropipette was placed and held for 10 minutes to ensure vector diffusion, and then gently removed.

Next, MePV was injected with rAAV-CaMKII-GCaMP6f (PT-0119, BrainVTA, China), rAAV-CaMKII-hM3Dq-mCherry (PT-0049, BrainVTA, China), rAAV-CaMKII-hM4Di-mCherry (PT-0017, BrainVTA, China), rAAV-CaMKII-mCherry (PT-1167, BrainVTA, China), AAV-CaMKII-ChR2-GFP (PT-3315, BrainVTA, China), or AAV-CaMKII-GFP (PT-1579, BrainVTA, China) 50 nl for each microinjection, Brain MePV According to the Franklin and Paxinos Mouse Brain Atlas, the coordinates were calculated from bregma (3rd edition). The skin was stitched using sterile medical sutures, and the mice were allowed to recuperate for a week. Behavioral studies were carried out three weeks following virus injection, after which all animals were subjected to histological examination to confirm the site of viral transduction. Any cases where viral transduction occurred outside of the MePV area were omitted from the analysis. All data in which the virus injection site was accurate should be retained, unless no virus expression, inaccurate virus injection site or virus expression exceeded the MePV brain region was found.

EEG-EMG recordings and analysis

Mice were implanted with electroencephalogram (EEG) and electromyogram (EMG) electrodes for polysomnographic recordings around two weeks following the virus injection. Four tiny craniotomy holes were made in the frontal area and cerebellum (bregma coordinates: ML = -1.50 mm; AP = +1.50 mm. cerebellum: reference electrode) to track EEG activity. Holes were carefully drilled in the cortex, and then four stainless steel tiny screws (1.2 mm head, 1.5 mm length) were placed therein. Two stainless steel wire electrodes were implanted into the dorsal neck musculature to measure nuchal EMG activity. Prior to this, each electrode wire was attached to a 6-pin connection. The bare skull was entirely covered with a small coating of Super-Bond C&B dental cement and the second coat of regular dental cement, and implants for the EEG-EMG recording equipment were fastened in place. Mice were allowed to recuperate for a week following the implantation of an EEG-EMG recording device. After at least three days of environment adaptation, EEG and EMG were recorded in separate soundproof recording rooms.

The EEG-EMG signals were amplified using sleep recording software, band-pass filtered (EEG: 0.1-30 Hz, EMG: 50-150 Hz), and then digitalized at 2 kHz (medusa, Bio-signal technologies, China). The resulting polygraphic recording signal was automatically evaluated using the sleep analysis program (Sirenia sleep 2.0.6, Pinnacle Technology, Inc.) based on the spectral fingerprints of EEG-EMG waveforms. States were categorized during a series of independent, 4 s epochs. Desynchronized, low-amplitude EEG cycles and increased EMG activity with phasic bursts were used to characterize wakefulness. NREM sleep was described as being synchronized, with high amplitude and low frequency

(0.5-4 Hz, delta) EEG activity and decreased EMG activity relative to awake and not having any phasic bursts. Theta rhythm (4–9 Hz) was used to identify REM sleep as well as the absence of any EMG activity. Microarousals were identified as transient disruptions (> 4s and 10s) in NREM sleep that were marked by sudden changes in theta and delta power activity in the EEG as well as the simultaneous emergence of high levels of EMG activity against a background of low muscle activity. A total of 5 and 4 NREM sleep epochs had to occur before and after a micro-arousal, respectively. Microarousals were recorded as "brief awakenings" and tallied as wakefulness because of their spectral characteristics. Scores were examined visually and manually adjusted where necessary. Delta (0.5–4 Hz), Theta (4–9 Hz), and Alpha relative bins were added to assess the power in certain frequency ranges (10-20 Hz). EEG power spectra were examined, 3 hours after CNO or saline injection, to assess the impact of the chemogentic activation of the MePV on power spectra for sleep-wake states.

Fiber photometry

For fiber photometry tests, a fluorescence photometer was coupled to the implanted optical fiber (Thinker Tech, China), and then a beam of 488 nm continuous laser light was briefly produced by a solid-state laser and reflected off a dichroic mirror to record GCaMP fluorescence data. Laser power was adjusted to a low setting at the optical fiber's tip to reduce GCaMP bleaching (405: 0.01-0.02 mW; 473: 0.03-0.05 mW). The same fiber was used to collect GCaMP fluorescence. Here, the light was directed through a dichroic lens with a GFP emission filter and detected by a photoreceiver. Using an avalanche photo-diode, the fluorescence was then detected and converted to electrical impulses. The output signals were amplified and digitalized at 2 kHz using the bespoke LabVIEW software. To calculate the GCamP6m signal, the relative fluorescence changes of △F/F were calculated to determine the Ca^{2+} signal as follows: △F/F= (F -F0) / F0. Both F and F0 were the corrected signal value excluding the noise value. The F0 was the average value of baseline value at the reference time point (before the event stimulus) and F was the fluorescent signal collected during the event stimulus. A specially created MATLAB algorithm was used to determine the average △F/F values. A Ca^{2+} transient's amplitude was considered a signal if it exceeded three times the noise band's standard deviation.

Histology

Mice were removed from the chamber and injected CNO or (only in c-Fos staining). After 30-60 mins ,mice were anaesthetized using 50 mg/kg i.p. sodium pentobarbital followed by a transcardial infusion of 30 ml ice-cold normal saline (NS, 0.9% sodium chloride) and 50 ml ice-cold 4% paraformaldehyde (PFA) in PBS. Next, their brains were carefully removed from the skull and postfixed overnight in 4% PFA, then dehydrated in 30% sucrose at 4 °C until they sank to the bottom of the tube. Brain tissues were sectioned into 40 nm coronal slices using a microtome (CM 1900, Leica), then stored in PBS at 4 °C until immunohistochemistry.

c-Fos staining: Sections were soaked in a blocking solution (Beyotime) and incubated for 20 min at room temperature (22±2 °C), and rinsed three times in PBS for 10 min each. Next, the sections were incubated for 24 hours with an anti-rabbit c-fos antibody (1:1000, Cell Signaling Technology) in a blocking solution at 4 °C. The sections were again washed three times in PBS (5-10 mins each), then incubated at room temperature with the following secondary antibodies for 90 minutes; Alexa Flour 488 donkey anti-rabbit IgG and Alexa Flour 568 donkey anti-rabbit IgG (1:500, Thermo Scientific). Sections were washed 3 times with PBS (5-10 mins each), stained with DAPI, again washed three times in PBS (3 mins each), mounted onto glass microscope slides, dried, and coated with mounting material.

The sections were finally observed under Olympus BX53 fluorescent microscope or confocal microscopy (Leica STELLARIS 5 SR), and images were processed with ImageJ 1.52V software.

Sleep deprivation:

We injected the virus into bilateral MePV nuclei in mice. Two weeks after the virus injection, EEG-EMG electrodes were implanted onto the surface of the mouse's skull, small stainless steel screws for EEG were implanted into the skull above the forehead, and small wire rings were inserted into the neck muscles for EMG recording. After the surgery, mice were allowed to recover for 7 days.

Before the start of the experiment, mice were housed individually in a sleeping chamber for at least 48 h and freely consumed water and food to adapt to the new experimental environment. 6 h (from 3 p.m.-9 p.m.) of sleep EEG/EMG recording after the acclimatization was performed (on day 0). Sleep deprivation was achieved from day 1 to day 5 using an automated sleep deprivation system, which consisted of an automatic cylindrical cylinder (43, 44) (PVC cylinder, height: 60 cm, width: 50 cm, weight: 5 kg), a small slow motor and a metal bar (45 cm) connected to the small motor. To induce sleep deprivation, the bar was continuously rotated with a motor at a speed of approximately 3 rpm and randomly reversed in the direction of rotation to prevent subjects from gaining short sleep periods by adapting to the rotation pattern. A trained experimenter visually verified that the wooden bar was spinning at all times from 9 a.m. to 1 p.m. and mice did not have any chance to sleep. 6 hours of EEG/EMG recording was performed immediately after the sleep deprivation. On day 6, the hM4D-CNO group was injected intraperitoneally with 1 mg/kg CNO and subjected to the elevated plus maze test and the open field test. At the end of the experiment, mice were returned to the sleep chamber for 6 hours of sleep EEG/EMG recordings. Meanwhile, the control group was injected intraperitoneally with 0.2ml/20g saline.

Electrophysiological analysis

Mice were anaesthetized with ketamine/xylazine (Sigma, 100/20 mg/kg, i.p.) as previously described (41, 42), their brains were taken out and immediately chilled using a modified artificial cerebrospinal fluid (ACSF) containing 220 mM sucrose, 1.3 mM $CaCl_2$, 2.5 mM KCl, 1 mM $NaH2PO_4$, 2.5 mM $MgSO_4$, 10 mM glucose, and 26 mM $NaHCO_3$. Brain tissues (300 nm) were sectioned using a VT-1000S vibratome (Leica, Germany) and incubated at 34 °C with normal ACSF (in mM, 126 NaCl, 1 $MgSO_4$, 3 KCl, 1.25 $NaH2PO_4$, 2 $CaCl_2$, 10 glucose, and 26 $NaHCO_3$) to recover for 30 min at 34 °C followed by additional 1 h at 25 °C before recording. All solutions were saturated with 95% O_2/5% CO_2 (vol/vol). At 32-34 °C, regular ACSF was superfused into the recording chamber at 2 ml/min. The recording pipettes (3-5 M) included 140 K-gluconate, 10 Hepes, 10 NaCl, 0.5 $MgCl_2$, 0.2 EGTA, 4 Mg-ATP, 10 phosphocreatine, and 0.4 Na-GTP (pH 7.4, 285 mOsm). Voltage-clamp recordings at were made 70 mV to isolate EPSCs. In addition, action potentials were recorded by delivering currents into the neuron for one second at zero potential while in an I-clamp condition. Blue light stimulation was provided by a blue LED (470 nm, inper) attached to the microscope by a dual lamp housing adapter.

Supplementary Fig. 1

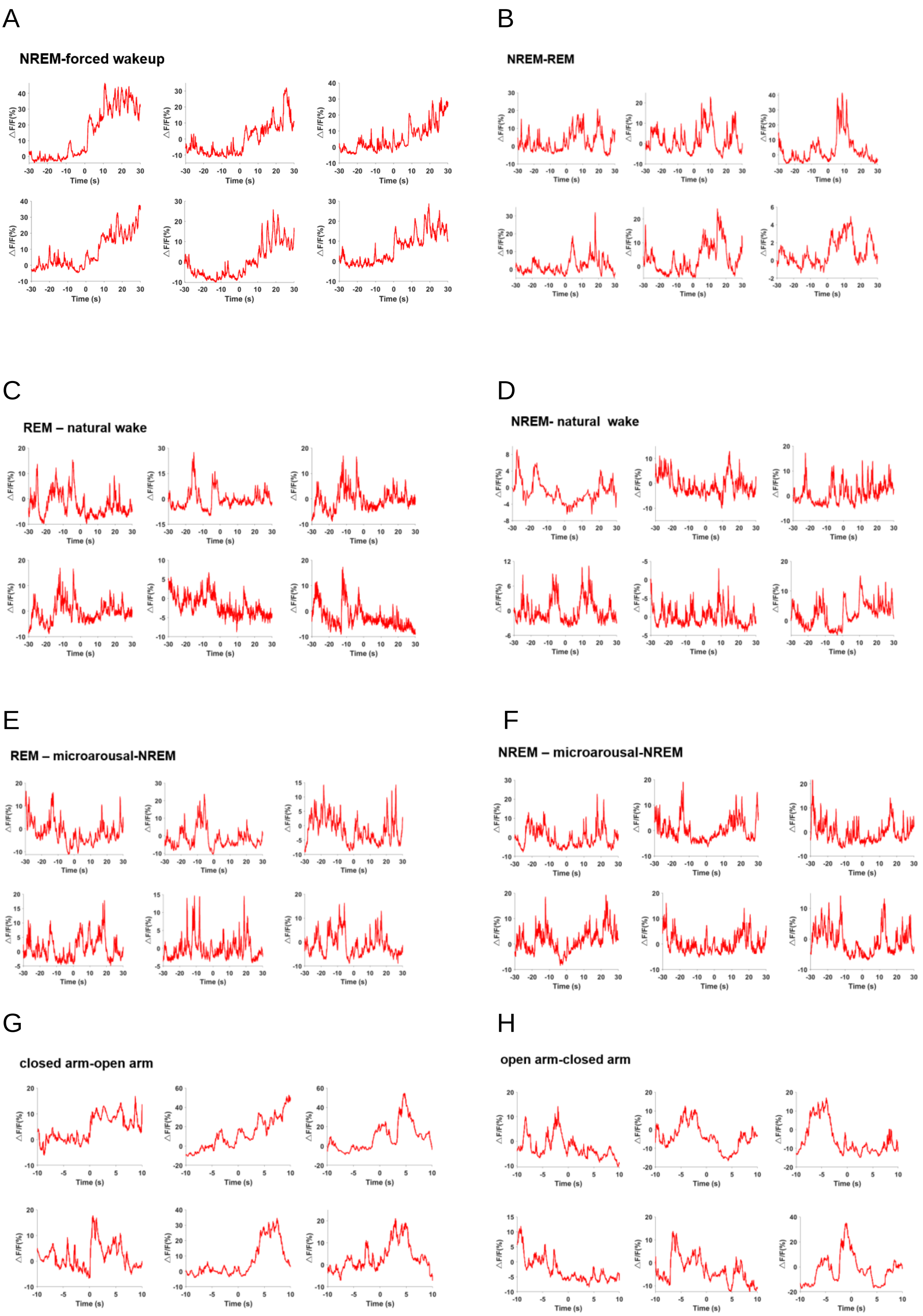

Supplementary Fig.1 In vivo MePV^{Glu} activity in different sleep pattern transitions and behavioral tests.

A, Representative Ca^{2+} signal traces of MePV^{Glu} neurons during the NREM-forced wakeup transitions; **B-D,** Representative Ca^{2+} signal traces of MePV^{Glu} neurons during NREM-REM transitions, REM-natural wake transitions and NREM-natural wake transitions. **E-F,** Representative Ca^{2+} signal

traces of MePV^{Glu} neurons indicated the representative transitions from REM to microarousal and from NREM to microarousal. **G-H,** Representative Ca^{2+} trace change of MePV^{Glu} neurons during elevated plus maze test. The representative traces were from 6 mice (once per animal).

Supplementary Fig. 2

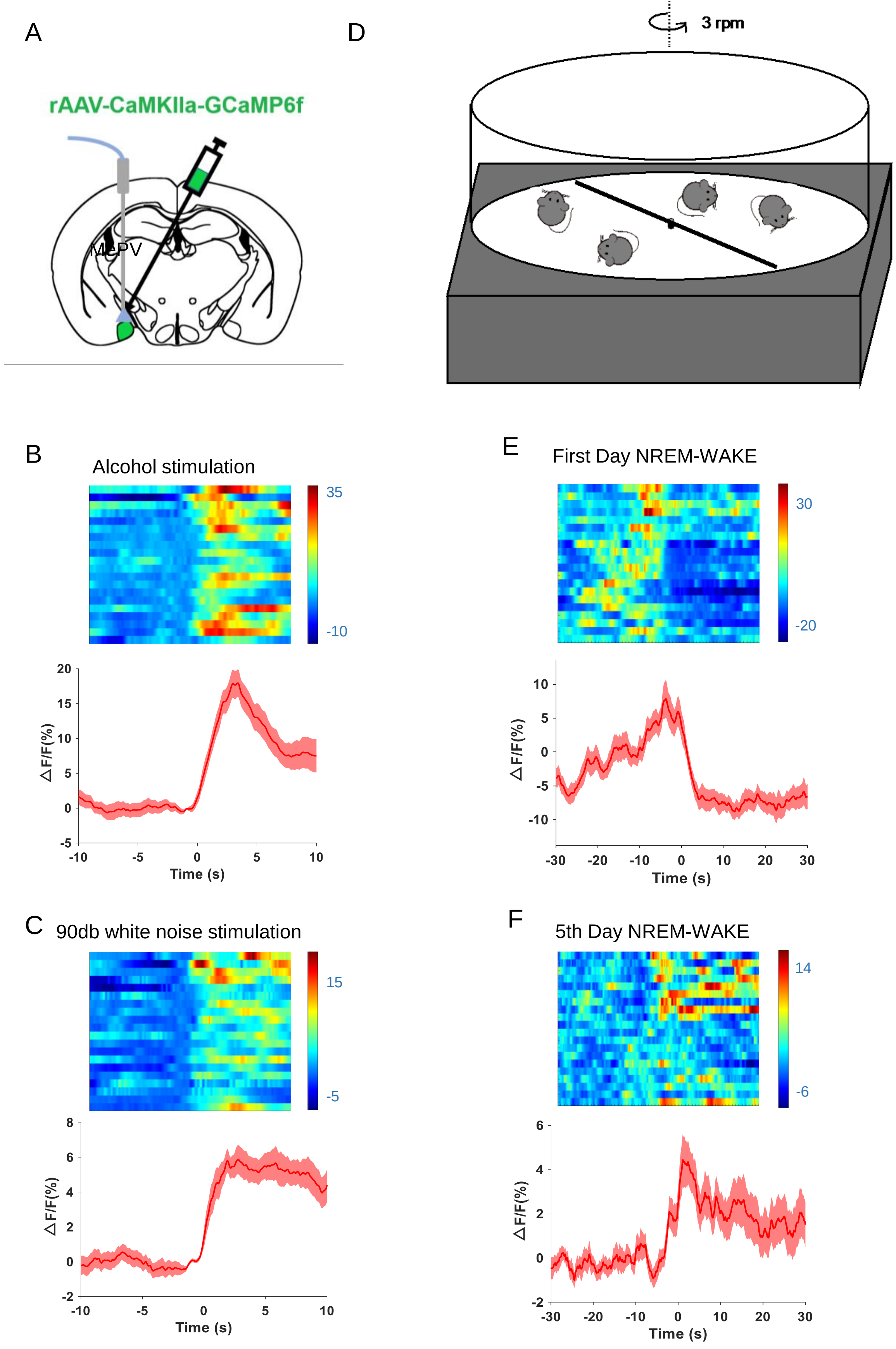

Supplementary Fig. 2 The activity of MePV^{Glu} neurons during sleep-forced wakeup transitions, and NREM-to-wake transitions after one day or 5th days of sleep deprivation.

A, The *in vivo* recording arrangement. **B,** The heat map for the Ca^{2+} signal of MePV glutamatergic neurons. The representative transitions (sleep-forced awakening by alcohol stimulation, n=5, 20 trails) of the changes in color-coded fluorescence intensity. **C,** The color-coded fluorescence intensity changes

of the representative shifts from the sleep to forced wakeup with 90 db white noise stimulation (n=5, 20 trails). **D,** Schematic diagram of the model of a sleep deprivation device. **E,** Changes in color-coded fluorescence intensity indicating the representative transitions from NREM to natural awakening on the first day of sleep deprivation (n=5, 20 trails). **F,** The representative shift in color-coded fluorescence intensity (n=5, 20 trails) depicting the transition from NREM to natural wake on the fifth day of sleep deprivation.

Supplementary Fig. 3

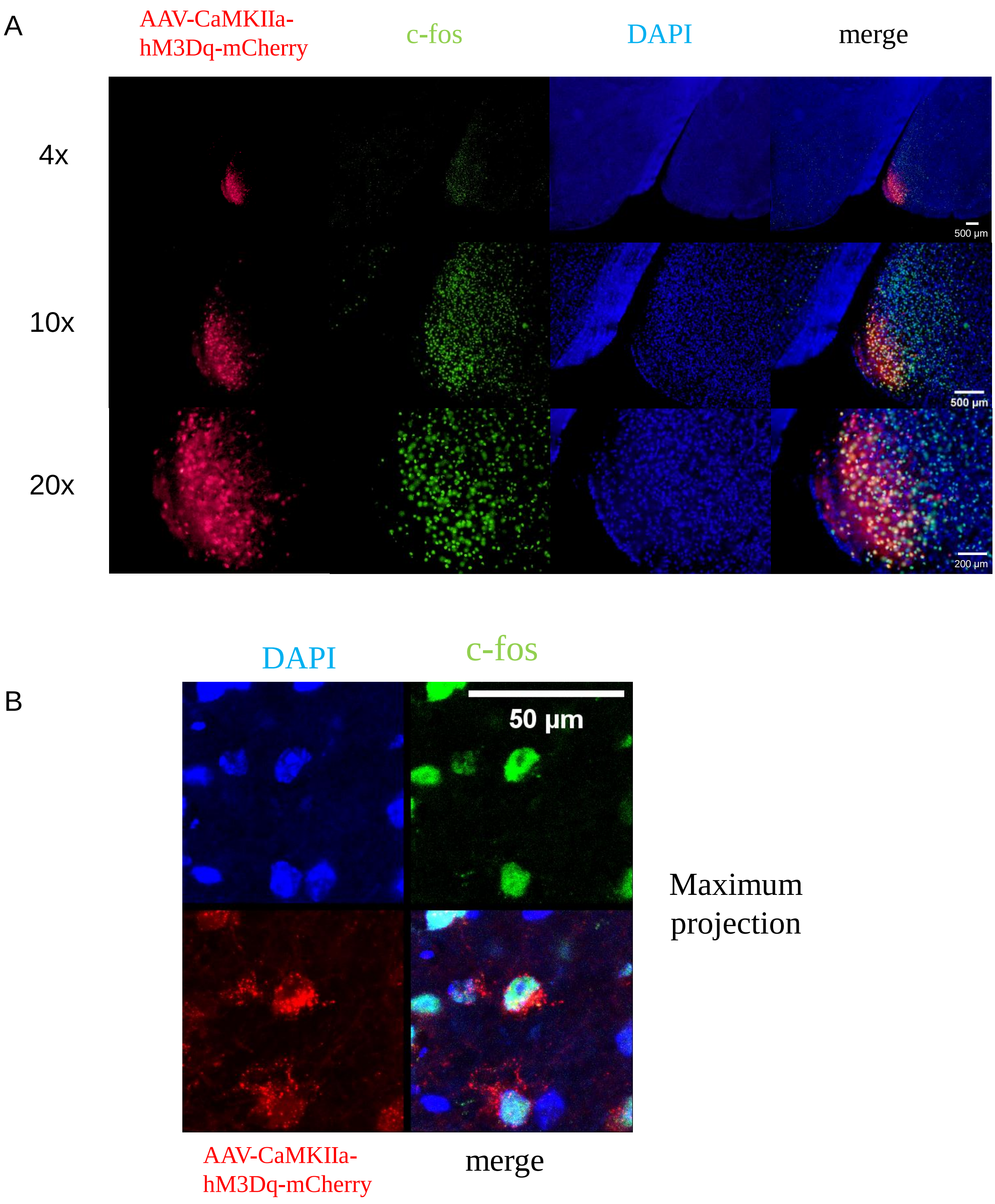

Supplementary Fig.3 Co-expression images of glutamic and cFos in MePV^{Glu} neurons.

A, Representative images (4x, 10x, 20x) showing the MePV colocalization of c-Fos (green), mCherry (red), and DAPI (blue) in *MePV*^{Glu} of hM3Dq mice treated with CNO by fluorescence microscope.

B, Maximum projection images (40x) showing the MePV colocalization of c-Fos (green), mCherry (red), and DAPI (blue) in *MePV*^{Glu} of hM3Dq mice treated with CNO by confocal microscopy.

Supplementary Fig.4

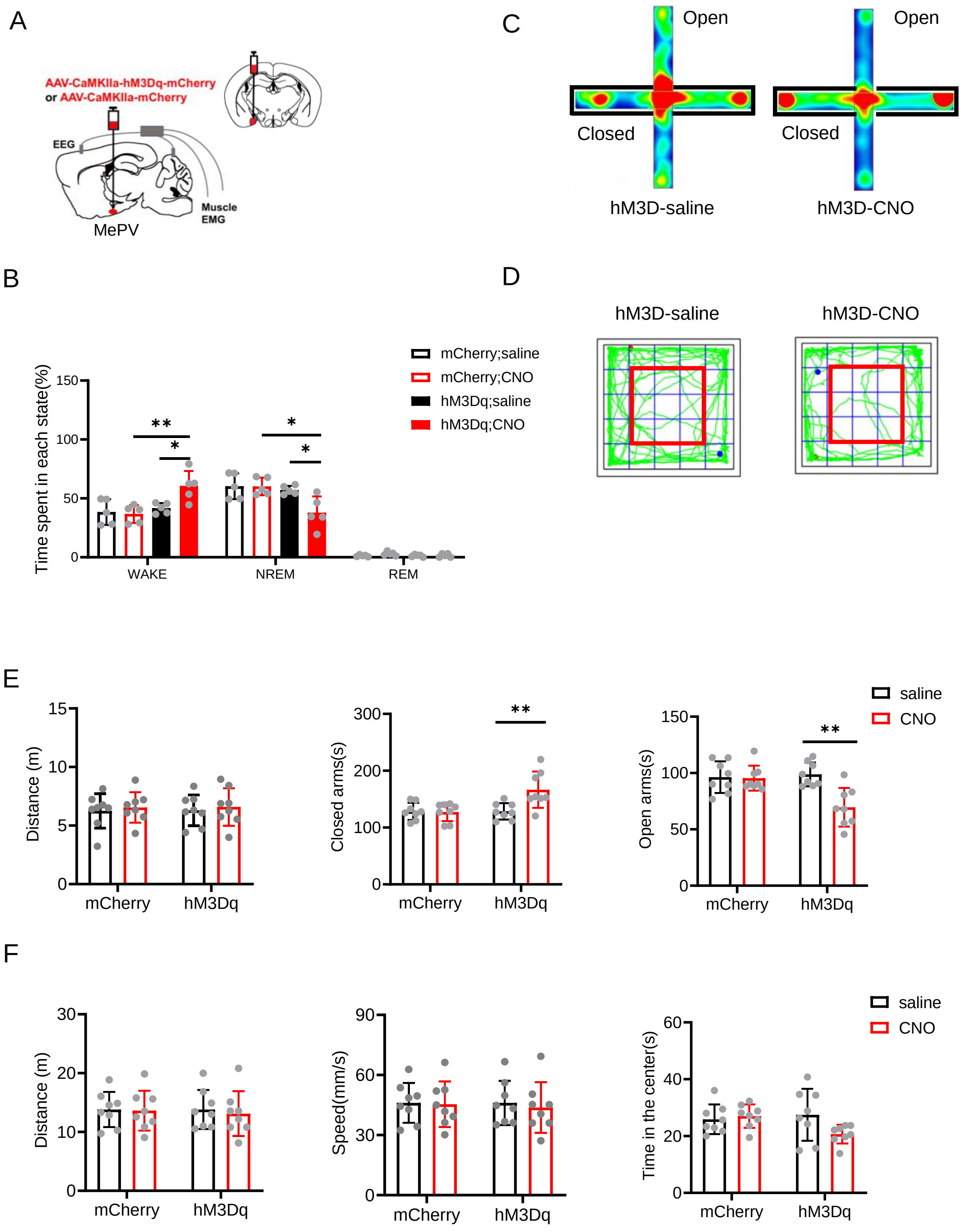

Supplementary Fig. 4 The effects of chemogenetic excitation of MePV^{Glu} neurons on wakefulness and anxiety-like behavior of female mice. A, Schematic diagram showing AAV-CaMKIIa-hM3Dq-mCherry/AAV-CaMKIIa-mCherry virus injection and EEG-EMG recordings. **B,** Percentages of time spent in each condition for MePV^{Glu}-hM3Dq female mice (n=5) and MePV^{Glu}-mCherry female mice (mCherry: n = 5 mice; hM3Dq: n = 5 mice) 3 hours after CNO

injection. **C,** Representative heatmaps of the elevated plus maze test. **D,** Representative track plots of open field test . **E,** The time spent on open/closed arms (left/middle). The distance of female mice (n=8) traveled on the elevated plus maze test(right). **F,** The time spent in the center zone and average speed traveled in the open field test(left and middle panel). The right panel represents the distance traveled in the open field test (right panel). *P<0.05, **P<0.01, and ***P<0.001. All comparisons were performed using one-way ANOVA and two-way ANOVA. Data are presented as mean ± SEM.

Supplementary Fig. 5

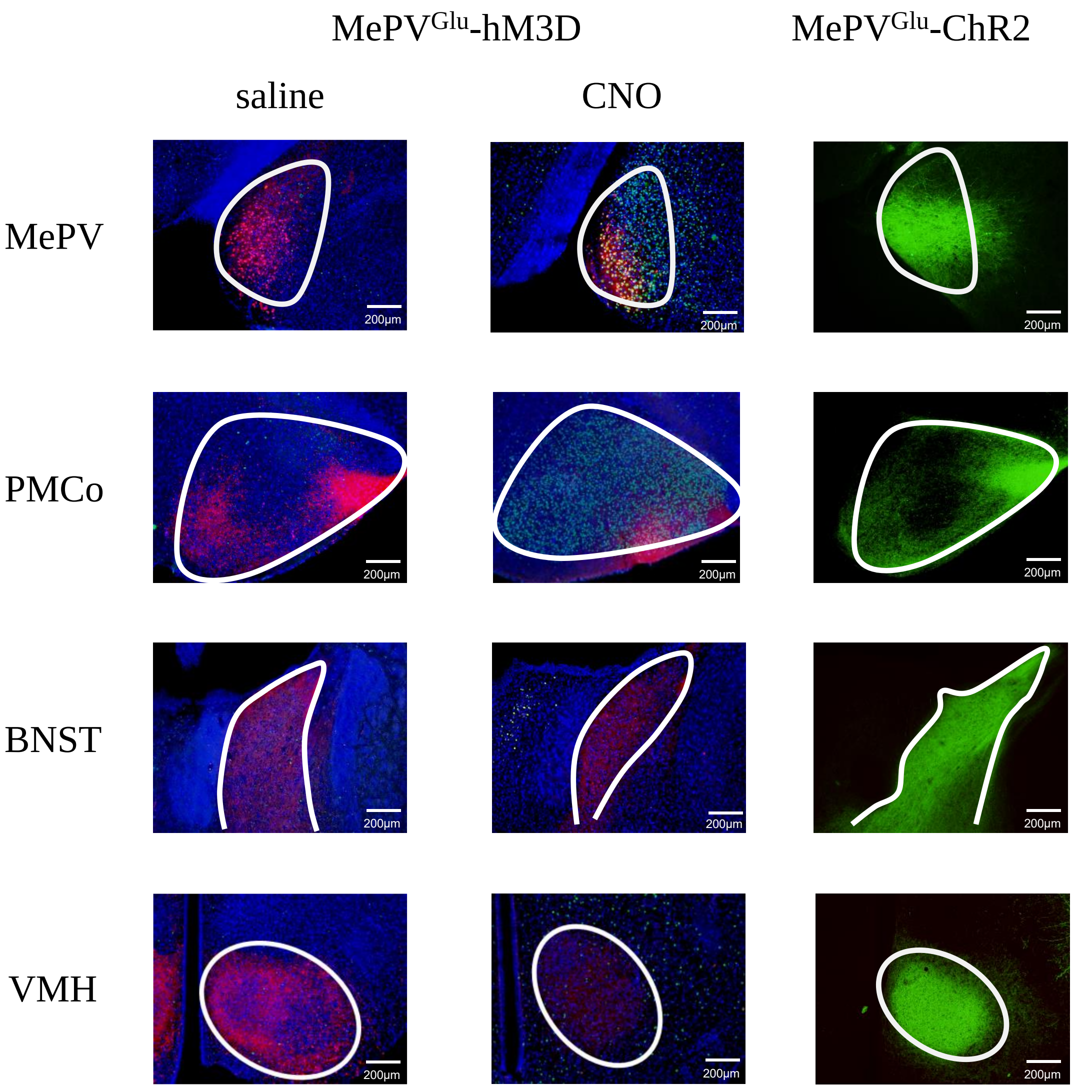

Supplementary Fig. 5 AAV and c-fos expression of MePV^{Glu} neurons and its projections before and after activation.

Left: Representative images showing the MePV and MePV's projections colocalization of c-Fos (green), mCherry (red), and DAPI (blue) in *MePV^{Glu}* of hM3Dq mice treated with saline.

Middle: Representative images showing the MePV and MePV's projections colocalization of c-Fos (green), mCherry (red), and DAPI (blue) in *MePV^{Glu}* of hM3Dq mice treated with CNO.

Right: Representative images showing the MePV and MePV's projections in *MePV^{Glu}* of ChR2 mice.

Supplementary Fig. 6

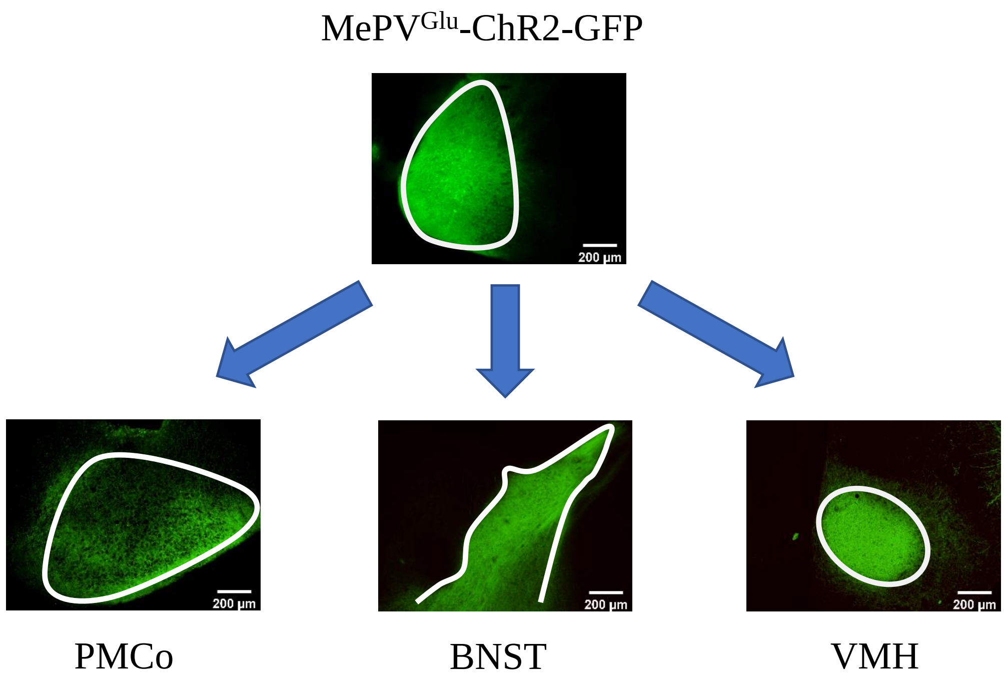

Left: Representative images showing the MePV and MePV's projections colocalization of c-Fos (green), mCherry (red), and DAPI (blue) in *MePV*^{Glu} of hM3Dq mice treated with saline.
Middle: Representative images showing the MePV and MePV's projections colocalization of c-Fos (green), mCherry (red), and DAPI (blue) in *MePV*^{Glu} of hM3Dq mice treated with CNO.
Right: Representative images showing the MePV and MePV's projections in *MePV*^{Glu} of ChR2 mice.

Supplementary Fig. 7

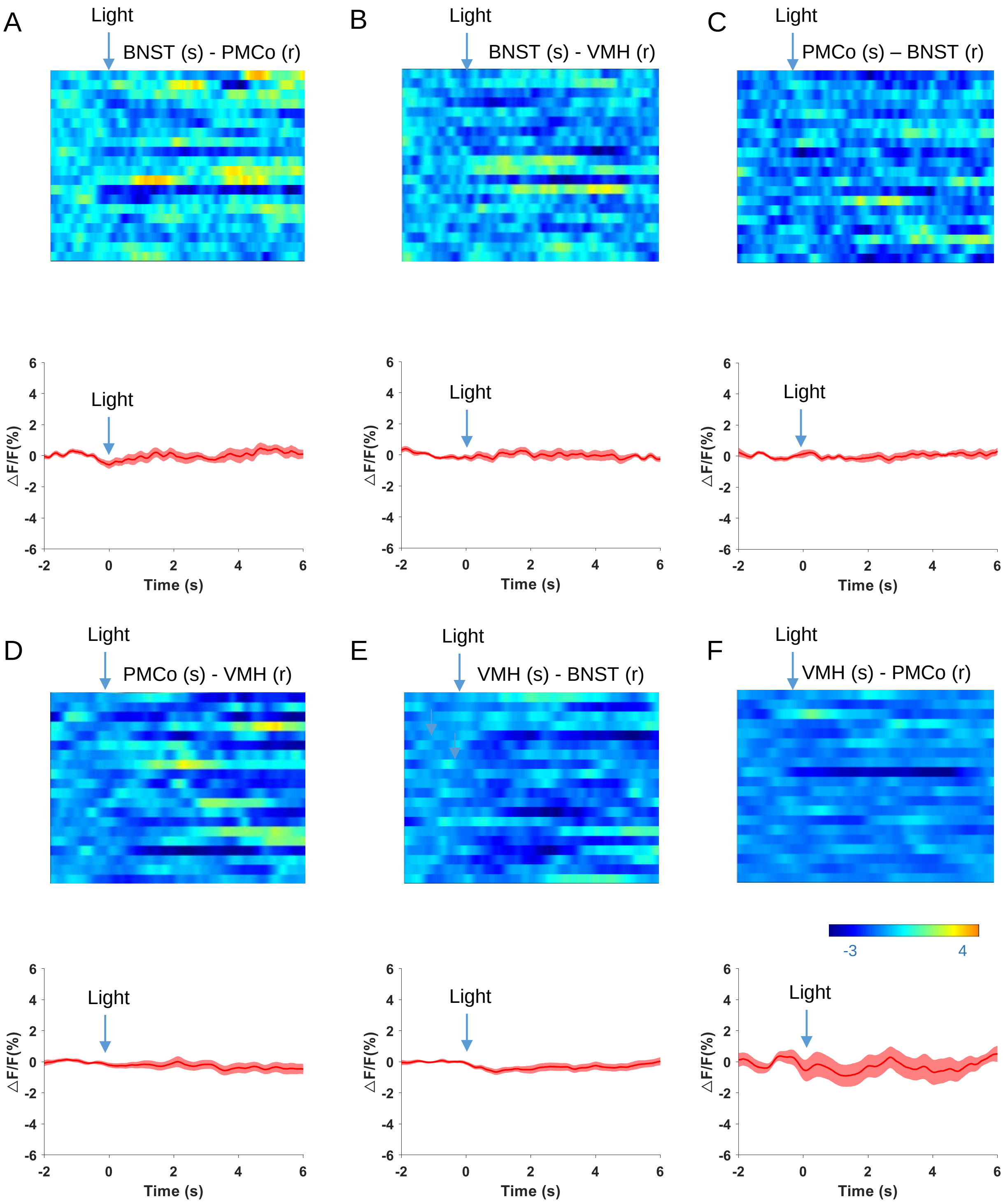

Supplementary Fig. 7 No interactions between different downstream projections of MePV^{Glu} neurons were found. The AAV-CaMKIIa-hChR2-mCherry virus was injected into the MePV and the rAAV-CaMKIIa-GCaMP6f virus was injected into the BNST, PMCo, and VMH to record the activity of glutamatergic neurons (r, recording; s: stimulating). **A,** Top panel, color-coded fluorescence intensity changes of the representative shifts from the light-off phase to the light-on phase (n=5, 20 trails). Down

panel, the mean value represented the average responses of PMCo neurons when activating MePV projecting terminals in the BNST. **B,** Top panel, color-coded fluorescence intensity changes of the representative shifts from the light-off phase to the light-on phase (n=5, 20trails). Down panel, the mean value represented the average responses of VMH neurons when activating MePV projecting terminals in the BNST. **C,** Top panel, color-coded fluorescence intensity changes of the representative shifts from the light-off phase to the light-on phase (n=5, 20 trails). Down panel, the mean value represents the average responses of BNST neurons when activating MePV projecting terminals in the PMCo. **D,** Top panel, color-coded fluorescence intensity changes of the representative shifts from the light-off phase to the light-on phase (n=5, 20 trails). Down panel, the mean value represents the average responses of VMH neurons when activating MePV projecting terminals in the PMCo. **E,** Top panel, color-coded fluorescence intensity changes of the representative shifts from the light-off phase to the light-on phase (n=5, 20 trails). Down panel, the mean value represents the average responses of BNST neurons when activating MePV projecting terminals in the VMH. **F,** Top panel, color-coded fluorescence intensity changes of the representative shifts from the light-off phase to the light-on phase (n=5, 20 trails). Down panel, the mean value represents the average responses of PMCo neurons, when activating MePV projecting terminals in the VMH.

Supplementary Fig. 8

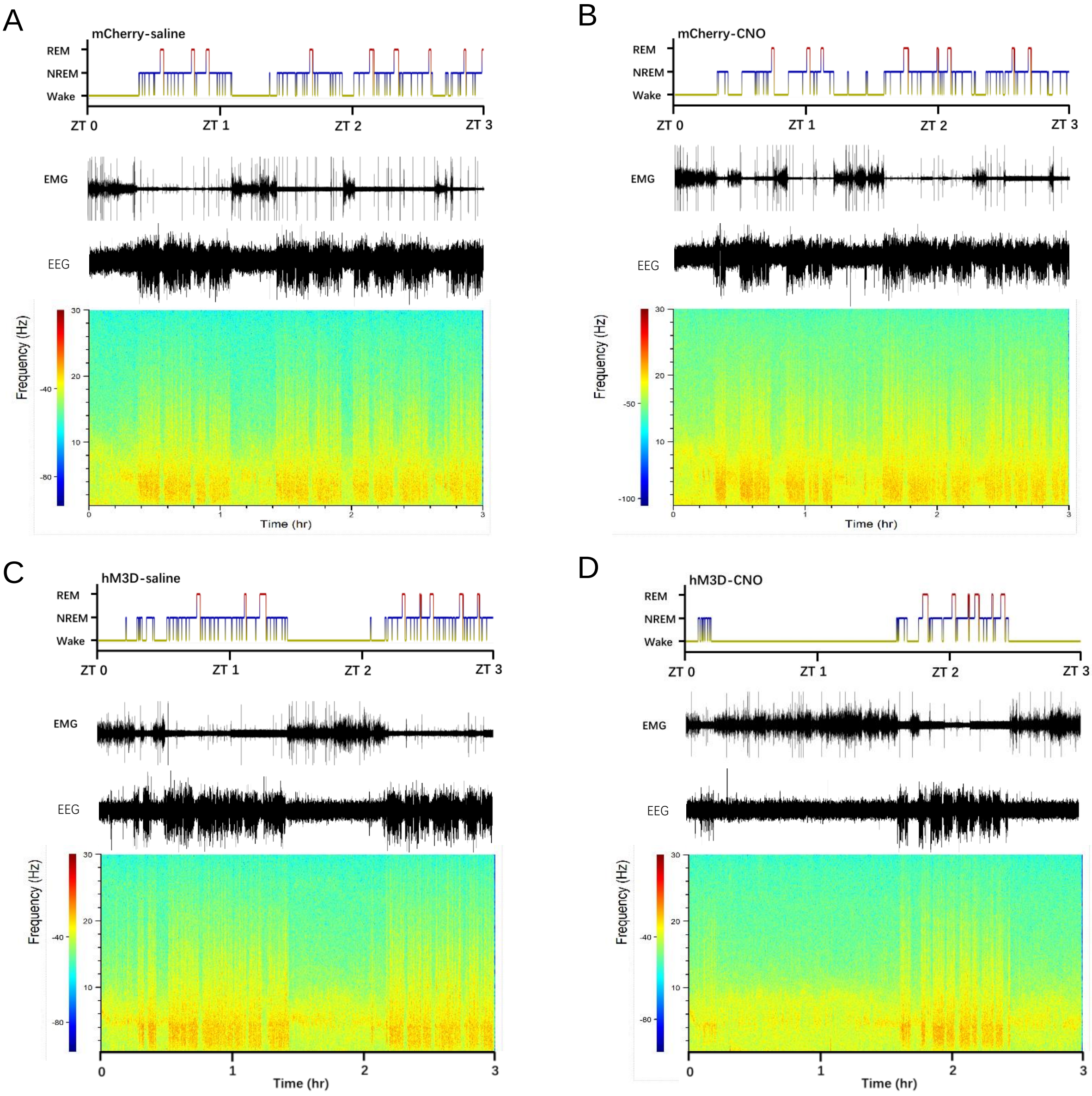

Supplementary Fig. 8 The effect of chemogenetic excitation of $MePV^{Glu}$ neurons on sleep-wake.

A, Examples of Hypnogram, EMG track, EEG track and EEG spectrogram from saline-injected *MePV-mCherry* mouse.

B, Examples of Hypnogram, EMG track, EEG track and EEG spectrogram from CNO injected *MePV-mCherry* mouse.

C, Examples of Hypnogram, EMG track, EEG track and EEG spectrogram from saline-injected *MePV-hM3Dq* mouse.

D, Examples of Hypnogram, EMG track, EEG track and EEG spectrogram from CNO injected *MePV-hM3Dq* mouse.

Supplementary Fig. 9

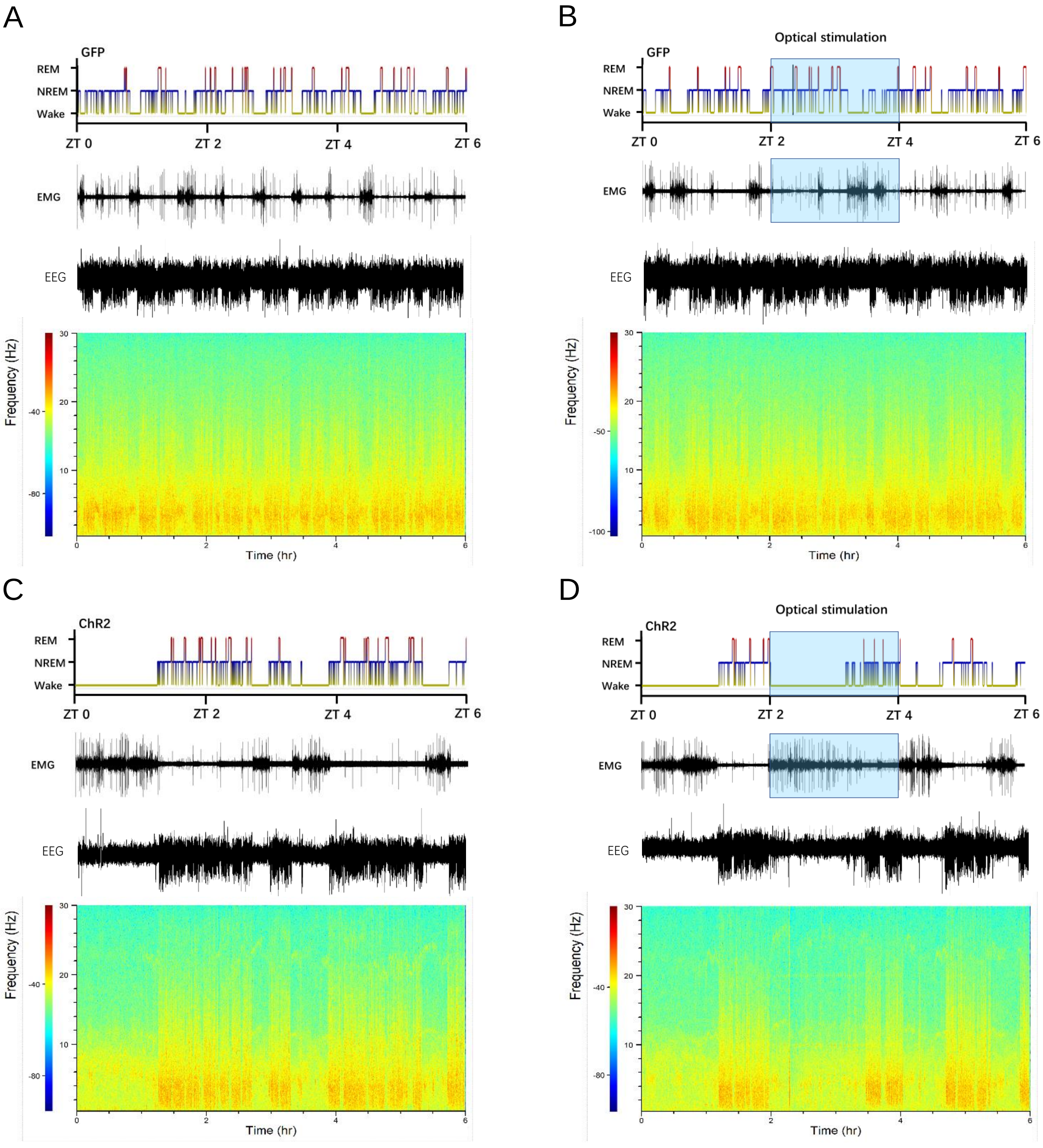

Supplementary Fig. 9 The effect of optogenetic excitation of $MePV^{Glu}$ neurons on sleep-wake.

A, Examples of Hypnogram, EMG track, EEG track and EEG spectrogram from no-intervention on $MePV^{Glu}$ GFP mouse.

B, Examples of Hypnogram, EMG track, EEG track and EEG spectrogram from blue light (n=470nm) effect on $MePV^{Glu}$ GFP mouse.

C, Examples of Hypnogram, EMG track, EEG track and EEG spectrogram from no-intervention on $MePV^{Glu}$ ChR2 mouse.

D, Examples of Hypnogram, EMG track, EEG track and EEG spectrogram from blue light (n=470nm) effect on $MePV^{Glu}$ ChR2 mouse.

Supplementary Fig. 10

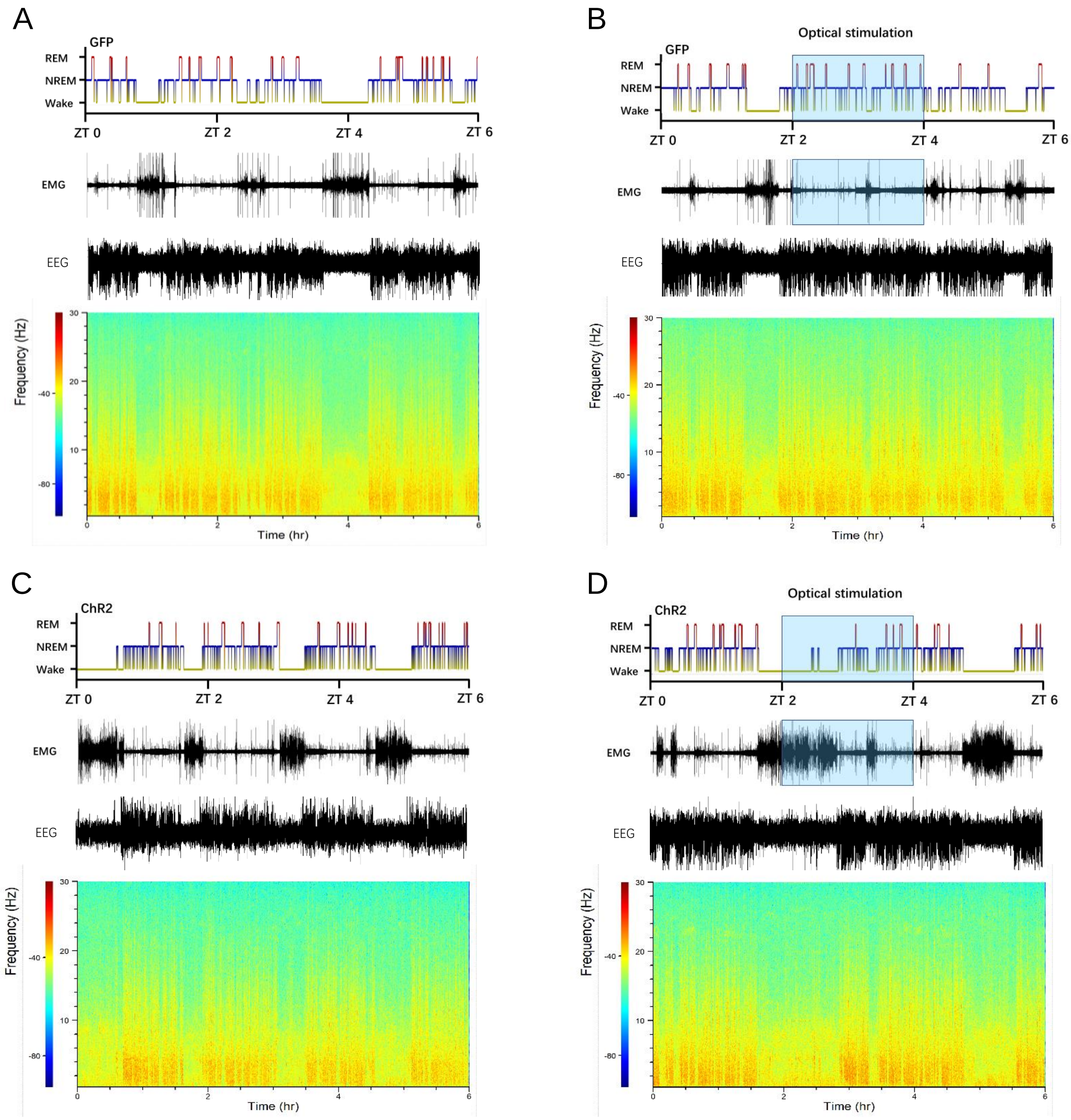

Supplementary Fig. 10 The effect of optogenetic excitation of $MePV^{Glu}$-PMCo circuit on sleep-wake.

A, Examples of Hypnogram, EMG track, EEG track and EEG spectrogram from no-intervention on *$MePV^{Glu}$-PMCo* GFP mouse.

B, Examples of Hypnogram, EMG track, EEG track and EEG spectrogram from blue light (n=470nm) effect on *$MePV^{Glu}$-PMCo* GFP mouse.

C, Examples of Hypnogram, EMG track, EEG track and EEG spectrogram from no-intervention on *$MePV^{Glu}$-PMCo* GFP mouse.

D, Examples of Hypnogram, EMG track, EEG track and EEG spectrogram from blue light (n=470nm) effect on *$MePV^{Glu}$-PMCo* ChR2 mouse.

Supplementary Fig. 11

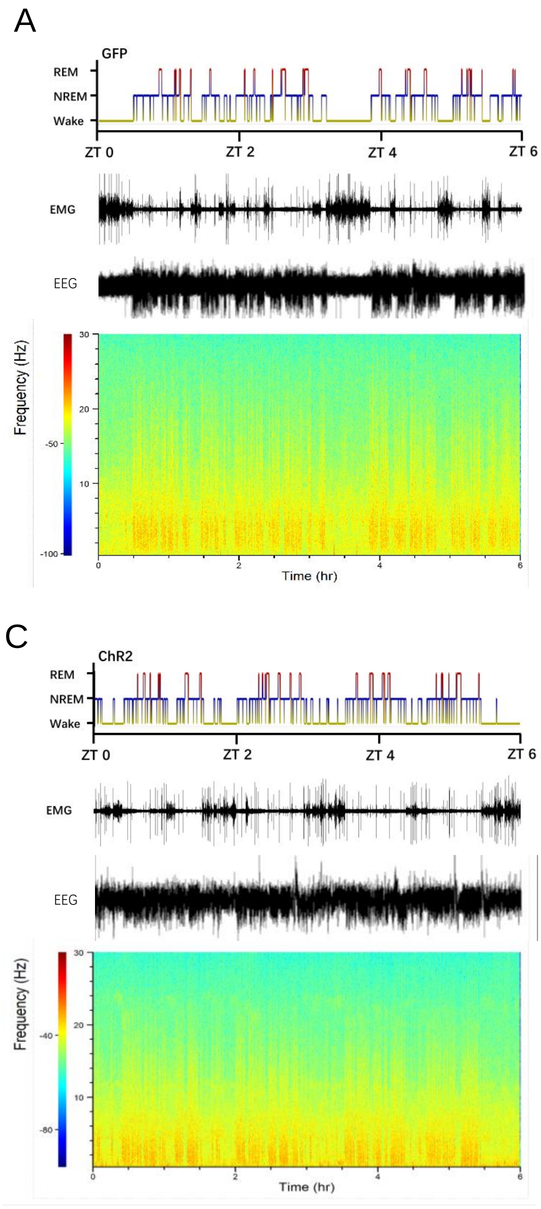

Supplementary Fig. 11 The effect of optogenetic excitation of $MePV^{Glu}$-BNST circuit on sleep-wake.

A, Examples of Hypnogram, EMG track, EEG track and EEG spectrogram from no-intervention on *MePV*^{Glu}*-BNST* GFP mouse.

B, Examples of Hypnogram, EMG track, EEG track and EEG spectrogram from blue light (n=470nm) effect on *MePV*^{Glu}*-BNST* GFP mouse.

C, Examples of Hypnogram, EMG track, EEG track and EEG spectrogram from no-intervention on *MePV*^{Glu}*-BNST* ChR2 mouse.

D, Examples of Hypnogram, EMG track, EEG track and EEG spectrogram from blue light (n=470nm) effect on *MePV*^{Glu}*-BNST* ChR2 mouse.

Supplementary Fig. 12

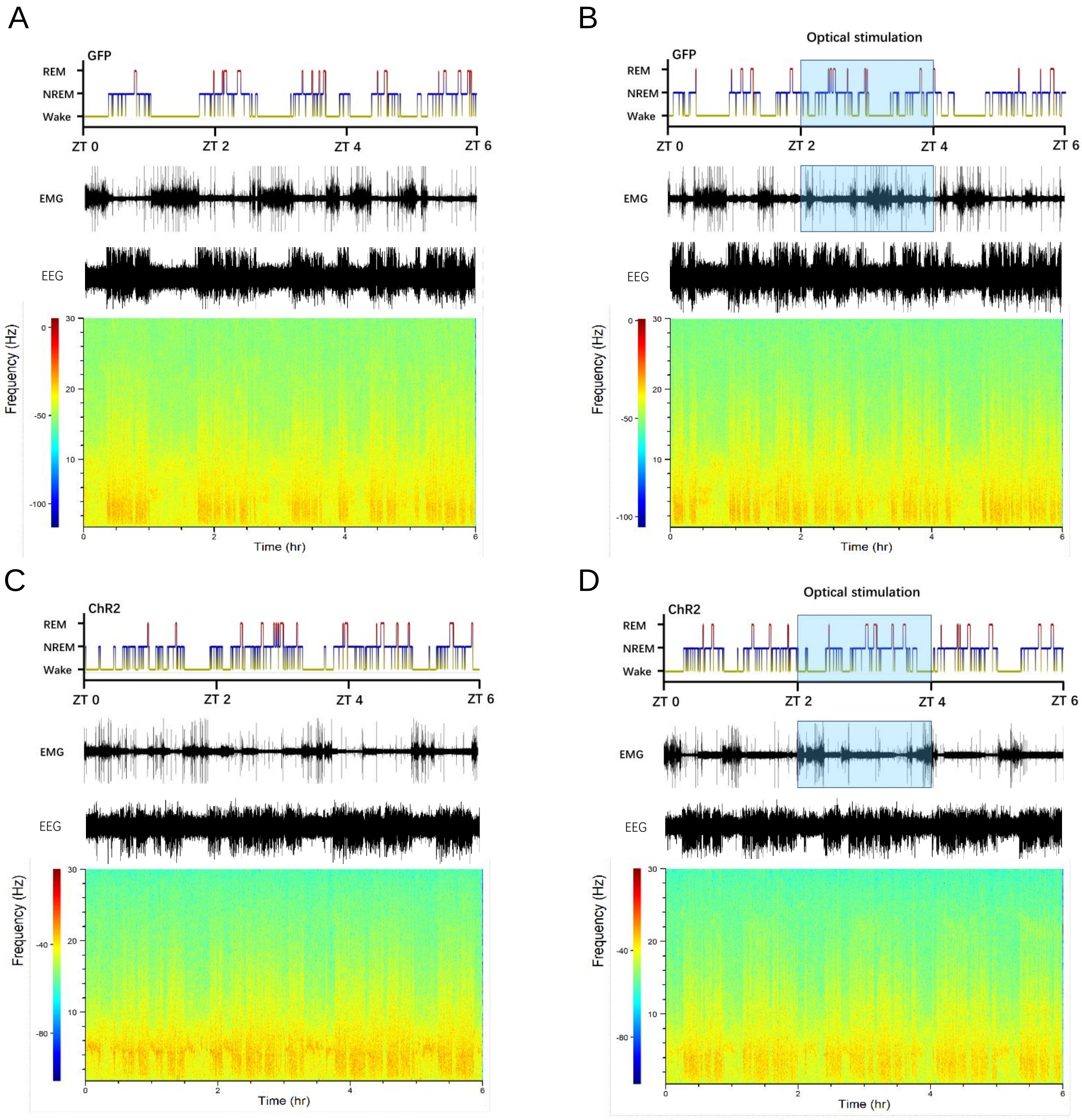

Supplementary Fig. 12 The effect of optogenetic excitation of MePV^{Glu}-VMH circuit on sleep-wake.

A, Examples of Hypnogram, EMG track, EEG track and EEG spectrogram from no-intervention on *MePV*^{Glu}*-VMH* GFP mouse.

B, Examples of Hypnogram, EMG track, EEG track and EEG spectrogram from blue light (n=470nm) effect on *MePV*^{Glu}*-VMH* GFP mouse.

C, Examples of Hypnogram, EMG track, EEG track and EEG spectrogram from no-intervention on *MePV*^{Glu}*-VMH* ChR2 mouse.

D, Examples of Hypnogram, EMG track, EEG track and EEG spectrogram from blue light (n=470nm) effect on *MePV*^{Glu}*-VMH* ChR2 mouse.

Supplementary Fig. 13

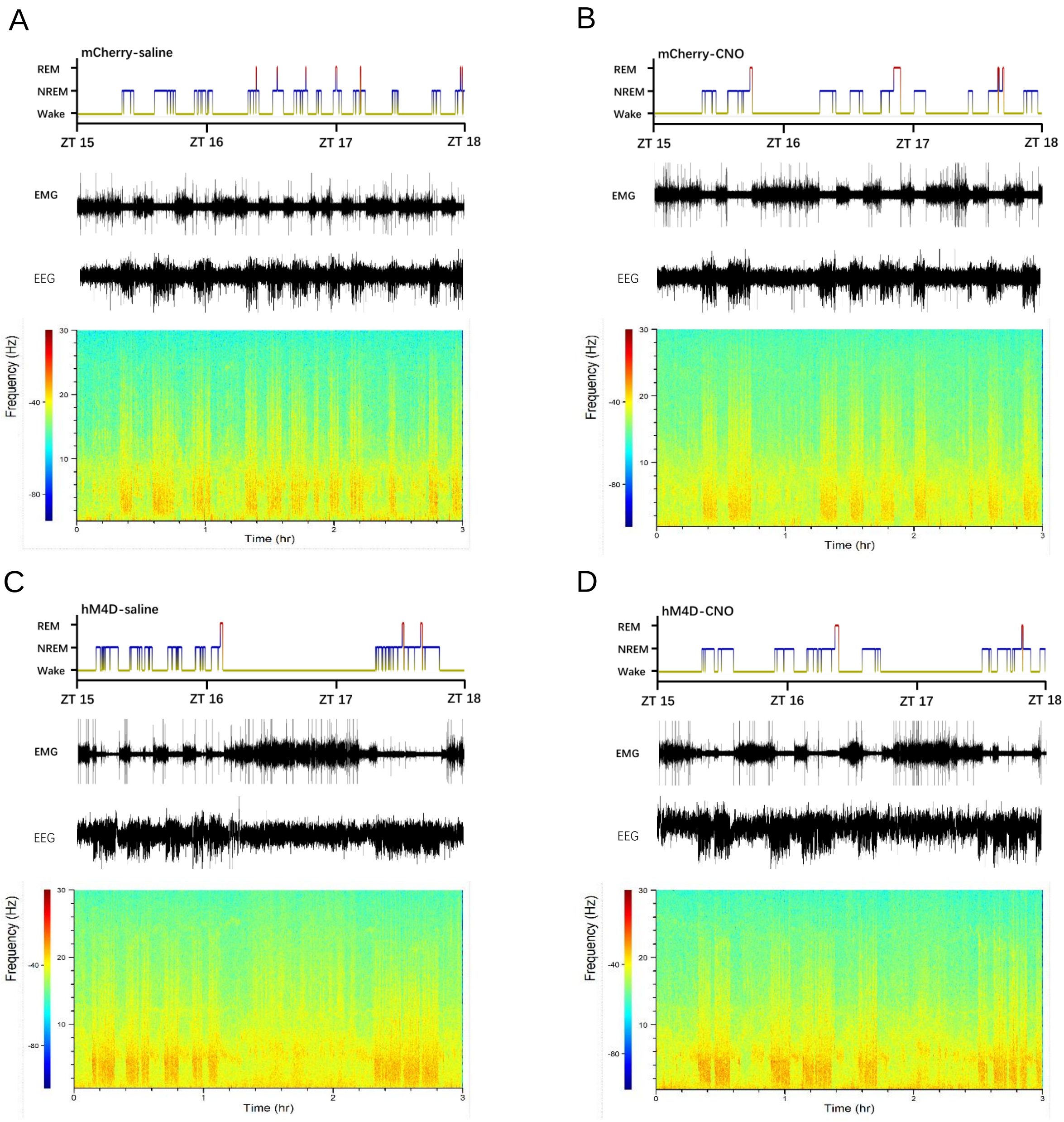

Supplementary Fig. 13 The effect of chemogenetic inhibition of $MePV^{Glu}$ neurons on sleep-wake.

A, Examples of Hypnogram, EMG track, EEG track and EEG spectrogram from saline-injected *MePV-mCherry* mouse.

B, Examples of Hypnogram, EMG track, EEG track and EEG spectrogram from CNO injected *MePV-mCherry* mouse.

C, Examples of Hypnogram, EMG track, EEG track and EEG spectrogram from saline-injected *MePV-hM4Di* mouse.

D, Examples of Hypnogram, EMG track, EEG track and EEG spectrogram from CNO injected *MePV-hM4Di* mouse.

Supplementary Fig. 14

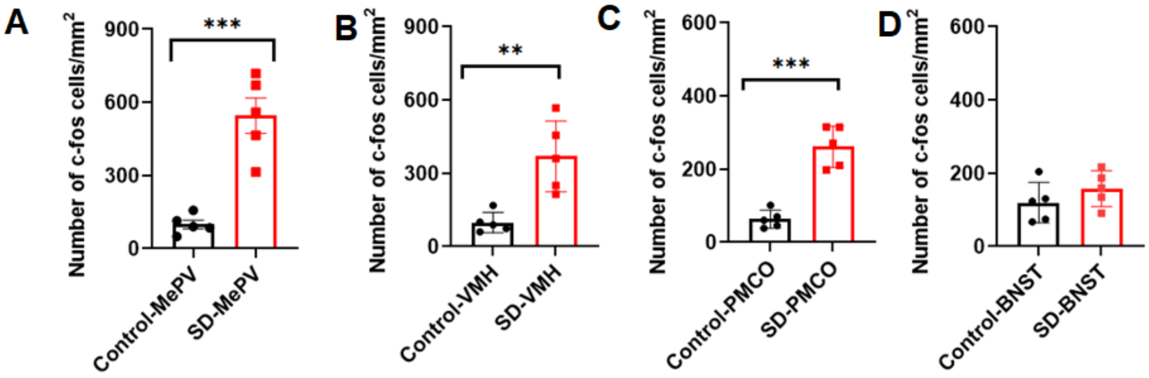

Supplementary Fig14. Quantification of c-Fos+ neurons in the MePV(A), VMH(B), PMCO(C), and BNST(D) and of Control and SD mice. Sleep deprivation significantly increased the number of c-Fos-expressing neurons in the MePV, VMH and PMCO area (8A, 8B and 8C). But sleep deprivation didn’t significantly affect the number of c-Fos-expressing neurons in the BNST area compared with the control group (8D).
